# Decoding the secrets of longevity: unraveling nutraceutical and miRNA-Mediated aging pathways and therapeutic strategies

**DOI:** 10.3389/fragi.2024.1373741

**Published:** 2024-03-28

**Authors:** Rania M. Salama, Nermin Eissa, Ahmed S. Doghish, Ahmed I. Abulsoud, Nourhan M. Abdelmaksoud, Osama A. Mohammed, Sherif S. Abdel Mageed, Samar F. Darwish

**Affiliations:** ^1^ Pharmacology and Toxicology Department, Faculty of Pharmacy, Misr International University, Cairo, Egypt; ^2^ Department of Biomedical Sciences, College of Health Sciences, Abu Dhabi University, Abu Dhabi, United Arab Emirates; ^3^ Department of Biochemistry, Faculty of Pharmacy, Badr University in Cairo (BUC), Cairo, Egypt; ^4^ Biochemistry and Molecular Biology Department, Faculty of Pharmacy (Boys), Al-Azhar University, Nasr City, Egypt; ^5^ Biochemistry Department, Faculty of Pharmacy, Heliopolis University, Cairo, Egypt; ^6^ Department of Pharmacology, College of Medicine, University of Bisha, Bisha, Saudi Arabia; ^7^ Pharmacology and Toxicology Department, Faculty of Pharmacy, Badr University in Cairo (BUC), Cairo, Egypt

**Keywords:** aging, age-related diseases, miRNAs, nutrition, nutraceuticals

## Abstract

MicroRNAs (miRNAs) are short RNA molecules that are not involved in coding for proteins. They have a significant function in regulating gene expression after the process of transcription. Their participation in several biological processes has rendered them appealing subjects for investigating age-related disorders. Increasing data indicates that miRNAs can be influenced by dietary variables, such as macronutrients, micronutrients, trace minerals, and nutraceuticals. This review examines the influence of dietary factors and nutraceuticals on the regulation of miRNA in relation to the process of aging. We examine the present comprehension of miRNA disruption in age-related illnesses and emphasize the possibility of dietary manipulation as a means of prevention or treatment. Consolidating animal and human research is essential to validate the significance of dietary miRNA control in living organisms, despite the abundance of information already provided by several studies. This review elucidates the complex interaction among miRNAs, nutrition, and aging, offering valuable insights into promising areas for further research and potential therapies for age-related disorders.

## Graphical Abstract

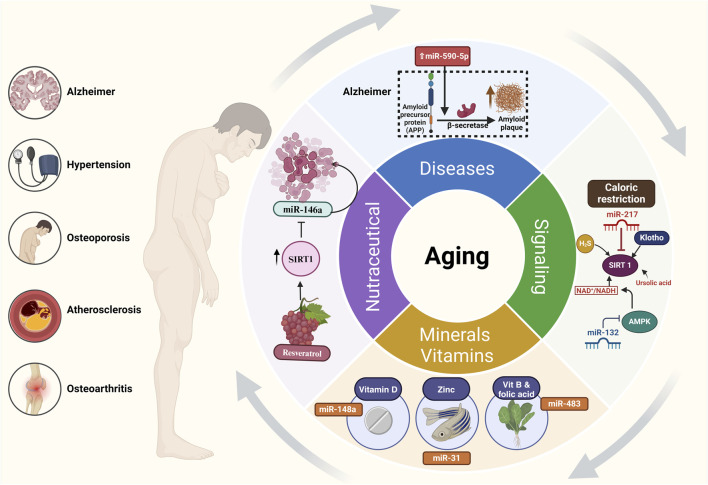

## 1 Introduction

MicroRNAs (miRNAs) are a type of short noncoding RNAs that have emerged as significant actors in epigenome modulatory actions. These little RNA molecules, usually composed of 18–25 nucleotides, implement their regulatory influence by attaching to messenger RNA (mRNA) molecules, resulting in the inhibition of protein synthesis or the breakdown of mRNA (Bartel, 2004; Lauria and Iacomino, 2021). Multiple biological processes have been linked to miRNAs, including cellular differentiation, development, and disease pathogenesis (Witwer, 2015).

In the complex system of gene regulation, miRNAs are essential for cellular homeostasis maintenance and for fine-tuning gene expression (Bartel, 2018). Aging and age-related disorders are linked to disruptions in miRNA expression and function. Cardiovascular disease, neurological illness, and cancer are only a few of the age-related ailments linked to miRNA dysregulation (Quinlan et al., 2017; Kinser and Pincus, 2020). New therapeutic intervention opportunities may arise from a better understanding of miRNAs’ roles in aging and age-related diseases (ElShelmani et al., 2021a; Matai and Slack, 2023b).

The aging process is only one of several health and disease outcomes that are profoundly impacted by nutrition (Leitão et al., 2022). Nutraceuticals are bioactive substances with dietary components that have the potential to regulate miRNA expression and function, according to emerging data (Kocic et al., 2019). The gene expression patterns linked to aging and age-related disorders can be influenced by foods such as macronutrients, micronutrients, and trace minerals, which in turn can change miRNA profiles (Beckett et al., 2014; Quintanilha et al., 2017b). In addition, nutraceuticals have demonstrated potential as treatment methods for age-related diseases by altering miRNA expression (Alnuqaydan, 2020; Ghosh et al., 2021).

A comprehensive literature search was carried out between July 2023 and October 2023 to find research papers and reviews related to the involvement of Nutraceutical and miRNA-Mediated Aging Pathways. The search was restricted to English items published in the past decade. We used the following electronic medical databases: Science Direct and PubMed. The search approach included a mix of the terms “miRNAs,” “microRNAs,” “Aging,” “nutrition,” and “nutraceuticals.” Furthermore, phrases like “gene expression regulation,” “molecular mechanisms,” and “pathogenesis” were included to guarantee a thorough search. The chosen publications underwent a comprehensive assessment, and pertinent data was retrieved. The key material encompassed in the article consists of the study design, sample size, miRNA profiling methodologies, experimental models, and conclusions about miRNA dysregulation and its influence on aging etiology and therapy. The collected data were combined to recognize recurring patterns, tendencies, and areas of limited understanding in the discipline. The quality and reliability of the studies were evaluated based on known criteria tailored to each research type, such as the Newcastle-Ottawa Scale for cohort studies and the Cochrane Collaboration’s tool for randomized controlled trials.

## 2 miRNAs biogenic pathways and function

### 2.1 Canonical and non-canonical miRNA biogenesis pathways

The role of non-coding RNAs (ncRNAs), which include miRNAs, in gene regulation is substantial across all eukaryotic organisms. Research on these little but powerful regulators has increased in recent years, illuminating their extensive effects on many different biological processes, as well as their extraordinary adaptability and complex regulatory networks (Cui et al., 2019; Abd El Fattah et al., 2022; El-Sheikh et al., 2022; Li et al., 2022; Bakr et al., 2023).

DNA sequences that are referred to as miRNA genes or clusters of genes that are either exclusively or cooperatively generated as miRNA molecules are the molecular antecedents of miRNAs. On the other hand, miRNAs can be discovered in the areas of non-translated or intron genes that are responsible for the production of proteins. Hereafter, we have outlined the canonical and non-canonical processes of biogenesis (Rodriguez et al., 2004; Olena and Patton, 2010).

**FIGURE 1:**
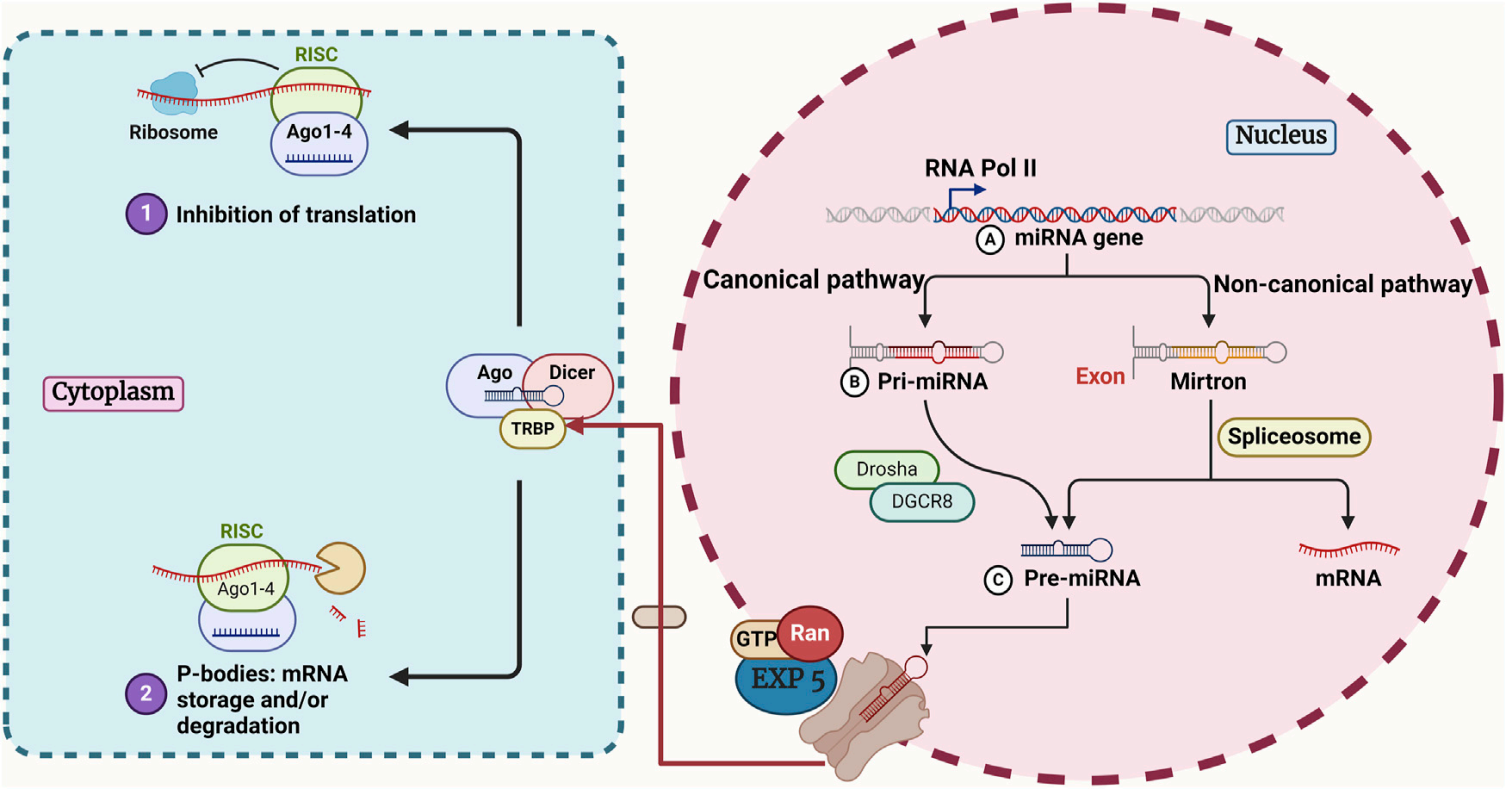
An overview of canonical and non-canonical pathways in miRNA biogenesis. In the canonical pathway, RNA Pol II transcribes the miRNA gene, and the microprocessor complex (Drosha and DGCR8) processes it to form a pre-miRNA. The non-canonical route is Drosha/DGCR8-independent and produces pre-miRNA directly from the entire intron. Both pathways converge as pre-miRNA is exported to the cytoplasm by EXP 5 and Ran-GTP. In the cytoplasm, Dicer processes pre-miRNA into a smaller double-stranded miRNA, which undergoes further cleavage to create a miRNA duplex. The duplex binds to Ago to form the RISC. The Ago-bound miRNA duplex unwinds, selecting either the 5p or 3p strand for the mature RISC complex. Ago, argonaute1; DGCR8, DiGeorge Critical Region 8; Dicer, an endoribonuclease enzyme that in humans is encoded by the DICER1 gene; Drosha, double-stranded RNA-specific endoribonuclease; EXP 5, Exportin-5; miRNA, microRNA; Ran, RAS-related nuclear protein; RISC, RNA-induced silencing complex; RNA Pol II, RNA polymerase II; TRBP, transactivation response element RNA-binding protein.

Within the canonical biogenesis process, the encoding of miRNA gene sequences by RNA polymerase II culminates in the primary miRNA (pri-miRNA) phase, which is the first step in the canonical pathway. The complex is responsible for transforming the pri-miRNA into the pre-miRNA, which is the precursor miRNA. The process of miRNA modification by Dicer begins with its transport to the cytoplasm via Exportin 5 (Denli et al., 2004; Newman and Hammond, 2010; Wei et al., 2021; Erturk et al., 2022). Single-stranded miRNAs that have reached maturity are responsible for directing the functional effector complex to modify complementary RNA targets (Figure 1) (Shang et al., 2023). The processing of some miRNAs will be carried out in a manner that is not deemed to be canonical. This particular area of study has been established with the support of a variety of various methods, such as transfer RNAs (tRNAs) derived miRNAs and miRtrons, in addition to small nucleolar RNAs (snoRNAs)-derived miRNAs and the independent approach that DICER has taken. The creation of these protocols allowed for the standardization of the many processes that are involved in the generation of miRNA (Ruby et al., 2007b; Babiarz et al., 2008; Ergin and Çetinkaya, 2022).

Transfer RNAs are an alternative source of non-canonical miRNAs. Angiogenin (ANG) or DICER uses the unique architecture of tRNAs as a substrate, cleaving the tRNA stem into fragments of tRNA-derived RNA (tDR) (Hasler et al., 2016). Also, some evidence suggests that AGO proteins can control gene expression in a manner analogous to miRNAs by loading certain tRNA segments (Abdelfattah et al., 2014). miRtrons are a kind of non-canonical pri-miRNAs that are encoded within the introns of coding genes. Like normal introns, miRtrons undergo early processing by nuclear splicing machinery, where they stabilize into hairpins with a shorter stem than conventional pri-miRNAs (Westholm and Lai, 2011). The debranching enzyme 1 (DBR1) is responsible for lariat-debranching rather than DROSHA/DGCR8 for these shorter hairpin configurations (Ruby et al., 2007a).

There is growing evidence indicating that some snoRNAs serve as a source for non-canonical miRNAs (Patterson et al., 2017). Notably, the processing of snoRNA-derived miRNAs and the stability of snoRNAs are influenced by key elements of the canonical miRNA biogenesis pathway, including DICER and DGCR8 (Langenberger et al., 2013). DGCR8, in conjunction with other proteins, could break down snoRNAs during their processing, therefore impacting the processing of miRNAs produced from snoRNAs (Macias et al., 2015). To mature, miRNAs may need the AGO2 slicer activity in a DICER-independent mode, which occurs when the stem-loop structure is too short to be cleaved by DICER (Cheloufi et al., 2010). Some of the benefits of small hairpin RNAs (shRNAs) processing that is not dependent on DICER include the ability to target genes in tumor cells that lack DICER and the preferential loading of small interfering RNAs (siRNAs) into AGO2, which improves RNAi (Herrera-Carrillo and Berkhout, 2017).

### 2.2 Function of miRNA

Even though miRNAs have been known for a considerable amount of time, it is only recently that their major role in an organism’s functioning has been understood. Cancer, skin issues, a broad range of lung disorders, neurological diseases, and other age-related diseases have all been the subject of extensive miRNA studies (Ugalde et al., 2011; Condrat et al., 2020; Semina et al., 2021). Evidence suggests that miRNAs can have a positive impact on a wide variety of critical biological processes. Inflammation, cell differentiation, and angiogenesis are all examples of such processes. Several complicated disorders have been linked to the improper regulation of non-coding RNAs. It is possible to affect cell function if the processing of these small but powerful regulators is disrupted (Abd El Fattah et al., 2023; El-Sheikh et al., 2023; Salman et al., 2023).

Research into complicated illnesses such as cancer, cardiovascular disease, and diabetes mellitus is facilitated by an increase in the quantity of data relevant to the interactions between miRNA and aging. These interactions apply to the process of aging. MiRNAs have also been shown to be responsible for the modulation of pathways that are involved in the sensing of nutrition (Ismail et al., 2019; Sohel, 2020; Abdelmaksoud et al., 2023).

To regulate a wide range of biological processes, miRNAs play an essential role. These regulatory molecules are likely to play a part in the etiology of inflammation and aging, and they might be utilized as therapeutic targets. There is little doubt about their involvement. Their actions are very context-dependent since miRNAs are engaged in post-transcriptional control of many messenger RNAs. Several factors impact these processes. These include the degree to which miRNAs interact with genes, the amount and affinity of miRNAs with their targets, the type of cell, the level of miRNA expression, and the concentration of miRNAs inside cells (Smith-Vikos and Slack, 2012; Quintanilha et al., 2017b; O'Brien et al., 2018). Epigenetics examines genetic control and acquired traits. Many epigenetic changes occur, including DNA methylation, histone modification, and miRNA channel silencing. (Al-Noshokaty et al., 2022; Rizk et al., 2022). The complicated mechanism that governs gene expression relies on miRNAs by the utilization of miRNA sponges, miRNA-Masking Antisense Oligonucleotides, or antisense oligonucleotides that specifically target miRNAs (AMOs) (Lima et al., 2018). In miRNA-sponge technology, mRNA molecules containing several target miRNA binding sites are expressed to act as a “sponge” or decoy for the target miRNAs (Ebert and Sharp, 2010). In other cases, instead of inhibiting the target miRNA, miR-Mask molecules shield the mRNA whose function is desired to retain. This technique is also known as BlockmiR, target protectors, or target site blockers (Wang, 2011; Beavers et al., 2015).

## 3 miRNAs and age-related diseases

### 3.1 Dysregulation of miRNAs in age-related diseases

One major risk factor for chronic illnesses in the elderly is aging. By attaching base pairs to their target mRNAs, miRNAs control the silencing of genes that occur post-transcription. Upon analyzing whole blood from healthy adults, recent studies found nonlinear variations in age-related miRNAs, with age having a greater influence than sex. In healthy aging, a transition in miRNAs to their 5′mature form was proved. With distinct disease biomarker sets for young and elderly patients, the inclusion of ill individuals highlighted pan-disease and disease-specific modifications in aging profiles (Meder et al., 2014; Fehlmann et al., 2020).

The altered expression of aging miRNA across various human and animal tissues has been confirmed through multiple studies (Smith-Vikos and Slack, 2012). Alterations in the expression of proteins, mRNA, and miRNA associated with aging and growth in the prefrontal cortex of rhesus macaques and humans have been noticed throughout their lives (Somel et al., 2010). Similar modifications in miRNA levels have been described in human skeletal muscle with aging (Drummond et al., 2011), as well as in bodily fluids like serum (Zhang H. et al., 2015). This lays the groundwork for recognizing the connection between disease and healthy aging as well as the creation of disease biomarkers particular to a person’s age (Table 1).

**TABLE 1:** The role of miRNAs in different age-related diseases.

Age-related disease	miRNA	Expression pattern	Target	Ref.
**Alzheimer’s disease**	let-7i-5p, miR-15a-5p	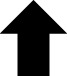	Apoptotic pathways	Sørensen et al. (2016), Poursaei et al. (2022)
	miR-29c-3p	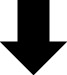		
	miR-142-5p	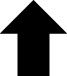	The G protein-coupled receptor BAI3	Fu et al. (2021)
	miR-122-5p, miR-210-3p, and miR-590-5p	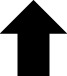	Amyloidogenesis	Mankhong et al. (2022)
	miR-342-5p	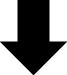	Prevent Aβ-mediated synaptic loss and regulation of some synaptic genes	Dakterzada et al. (2021)
	miR-1271	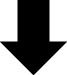	ALK and RYK	Majumder et al. (2021)
**Hypertension**	miR-181a, miR-663	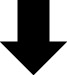	Renin mRNA	Marques et al. (2011)
	miR-143/145	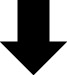	Angiotensin-converting enzyme	(Boettger et al., 2009; Kohlstedt et al., 2013)
	miR-21 and miR-221/222	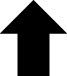	Proliferation of vascular smooth muscles and neointimal hyperplasia	(Chistiakov et al., 2015; Zhang et al., 2016)
	hcmv-miR-UL112	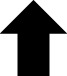	Interferon regulatory factor 1	Li et al. (2011)
	miR-505	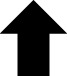	The migration and tube formation of endothelial cells	Yang et al. (2014b)
	miR-9, miR-126	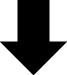	- Aldosterone-induced hypertrophic pathway - Angiogenic signaling and vascular integrity	Kontaraki et al. (2014)
**Heart Failure**	miR-18a-5p, −26b-5p, −27a-3p, −30e-5p, −106a-5p, −199a-3p, -652-3p, and −199a-3p	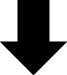	Cardiomyocyte proliferation, myocardial matrix remodeling, and cardiac hypertrophy	Ovchinnikova et al. (2016)
	miR-21, miR-214, and miR-27b	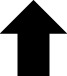	Myocyte hypertrophy and fibrotic process	Gholaminejad et al. (2021)
	miR-126 and -508-5p	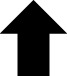	Angiogenesis pathway in endothelial progenitor cells dysfunction	Qiang et al. (2013)
	miR-30d	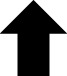	Apoptosis mediated by tumor necrosis factor and the downstream effector; mitogen-associated kinase 4	Melman et al. (2015)
	miR-155	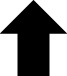	Inflammation and neovascularization	Wang et al. (2017a)
**Atherosclerosis**	miR-122	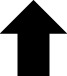	Plasma levels of total cholesterol	Huang et al. (2022)
	miR-30c, miR-33a	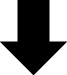 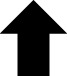	- LDL, and lesions of atherosclerosis - HDL-C	Yaman et al. (2021)
	miR-148a and miR-128-1	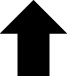	LDL and lipoproteins	Santos et al. (2021)
	miR-33	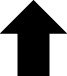	HDL and inflammatory genes ABCA1 and ABCG1	(Rayner et al., 2011; Goedeke et al., 2014); Zhang et al. (2022)
	miR-181b	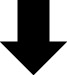	Endothelium inflammation	Sun et al. (2014)
	miR-27a/b	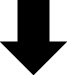	Cholesterol metabolism genes and homeostasis of macrophages within the plaque	Zhang et al. (2014)
	miR-34a	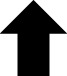	Inflammation, and macrophage cholesterol export	Xu et al. (2020)
	miR-133	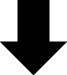	The uptake of oxidized LDL by foam cells	Gabunia et al. (2017)
	miR-302 and miR-26	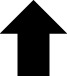	Foam cells development	Johnson (2019)
	miR-21-3p	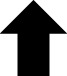	Vascular smooth muscle migration and proliferation	Zhu et al. (2019)
	miR-200c	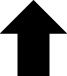	SIRT1, eNOS, and FOXO1	Magenta et al. (2018); Carlomosti et al. (2017)
**Osteoarthritis**	miR-483-5p, miR-149, -582-3p, −1227, −634, -576-5p, and −641	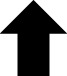 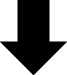	Cartilage function and SIRT1 pathway	Díaz-Prado et al. (2012)
	miR-34a	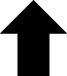	Cartilage breakdown	Tao et al. (2020)
	miR-146a	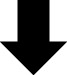	Apoptosis and autophagy of chondrocytes	Zhang et al. (2021)
	- miR-146a-5p and miR-34a-5p - miR-127-5p and miR-140-5p	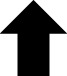 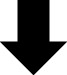	Mesenchymal stem cells and transforming growth factor-β	Liu et al. (2023)
	miR-124	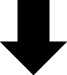	Chondrocyte apoptotic cascade, extracellular matrix breakdown, and synovial thickness	Shi et al. (2023)
	miR-107 and miR-143-3p	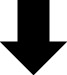	Protein translation, proliferation, and hypertrophy in chondrocytes	Balaskas et al. (2023)
**Osteoporosis**	miR-133a	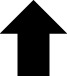	Osteoblastogenesis	Li et al. (2018b)
	miR-214	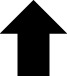	Osteoblast and osteoclast activities, bone metabolism	(Sadu et al., 2023; Wang et al., 2023)
	miR-138-5p	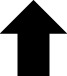	Microtubule actin cross-linking factor 1 and the differentiation of aged osteoblasts	Chen et al. (2022)
	miR-182	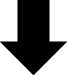	Forkhead box O1 and osteogenesis	Chen et al. (2019)
	miR-148a	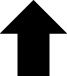	Osteoclastogenesis	(Tian et al., 2021; Pan et al., 2022)
**Macular Degeneration**	miR-885-5p, miR-486-5p and miR-626	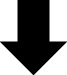 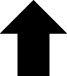	Apoptotic and neovascularization pathways	Elbay et al. (2019)
	miR-155 and miR-27a, miR-146a	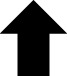	Genes implicated in the transforming growth factor-β route Nuclear factor Kappa B, tumor necrosis factor, and Toll-like receptors	Romano et al. (2017)
	MiRNA-7, miRNA-9-1, miRNA-23a/miRNA-27a, miRNA-34a, miRNA-125b-1, miRNA-146a	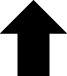	Complement factor H	(Hill et al., 2015; Pogue and Lukiw, 2018)
	miR-15/107 group, the miR-17∼92 cluster, miR-21, miR-132, miR-296, miR-378, and miR-519c	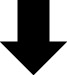	Angiogenesis and macular related-apoptosis	(Ren et al., 2017; Urbańska et al., 2022)
	miR-19a, miR-126, and miR-410	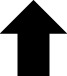	Regulate angiogenesis and retinal vascular development or Corneal neovascularization	ElShelmani et al. (2020)
	let-7a-5p, miRNA-17-5p, miRNA195-5p, miRNA26b-5p, and miRNA-30c-5p	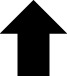	Apoptotic pathways	ElShelmani et al. (2021b)

Extracellular vesicles (EV) are considered fragments of dead or senescent cells secreted upon physiological or pathological processes (Misawa et al., 2020). EVs are used to send signals for exchanging information among cells via a complex packet of lipids, and proteins rich in ncRNAs and miRNAs. They are also known as exosomes or microvesicles and have an important role in aging (Tkach and Théry, 2016; Cooks et al., 2018).

### 3.2 Role of miRNAs in different age-related diseases

Our current knowledge of the significant role miRNAs perform in age-related disorders is not sufficiently comprehensive due to the varied nature of intracellular and extracellular miRNAs. Nonetheless, interesting research has been done that will help us grasp the fundamental causes of these diseases. Herein, we will discuss a growing number of publications about particular miRNAs and their involvement in different age-related disorders.

#### 3.2.1 miRNAs in Alzheimer’s disease

A fundamental neurodegenerative process linked to aging in individuals with Alzheimer’s, dementia, Huntington’s, and Parkinson’s disease patients is cognitive loss. Noncoding miRNAs have a major role in CNS, which has led to the discovery of promising novel clinical prospects for these illnesses, which lack current effective therapies (Abdelmaksoud et al., 2024).

The most frequent cause of dementia is Alzheimer’s disease (AD), which is characterized by a gradual degeneration of neurons and cognitive abilities (Bandakinda and Mishra, 2023). The let-7 miRNA family has been shown to have pro-apoptotic properties in the central nervous system and to control the proliferation and differentiation of neural stem cells. Numerous investigations have demonstrated that aberrant let-7 miRNA activity may contribute to the disease linked to dementia and cognitive decline via important neuronal signaling pathways (Fairchild et al., 2019). Sorensen et al. examined the blood and CSF of people suffering from AD and other forms of dementia in a different investigation (Sørensen et al., 2016). When comparing AD patients to healthy controls, they found 52 miRNAs in the CSF of nearly all patients, of which two (let-7i-5p and miR-15a-5p) were elevated and one (miR-29c-3p) was downregulated (Poursaei et al., 2022).

**FIGURE 2:**
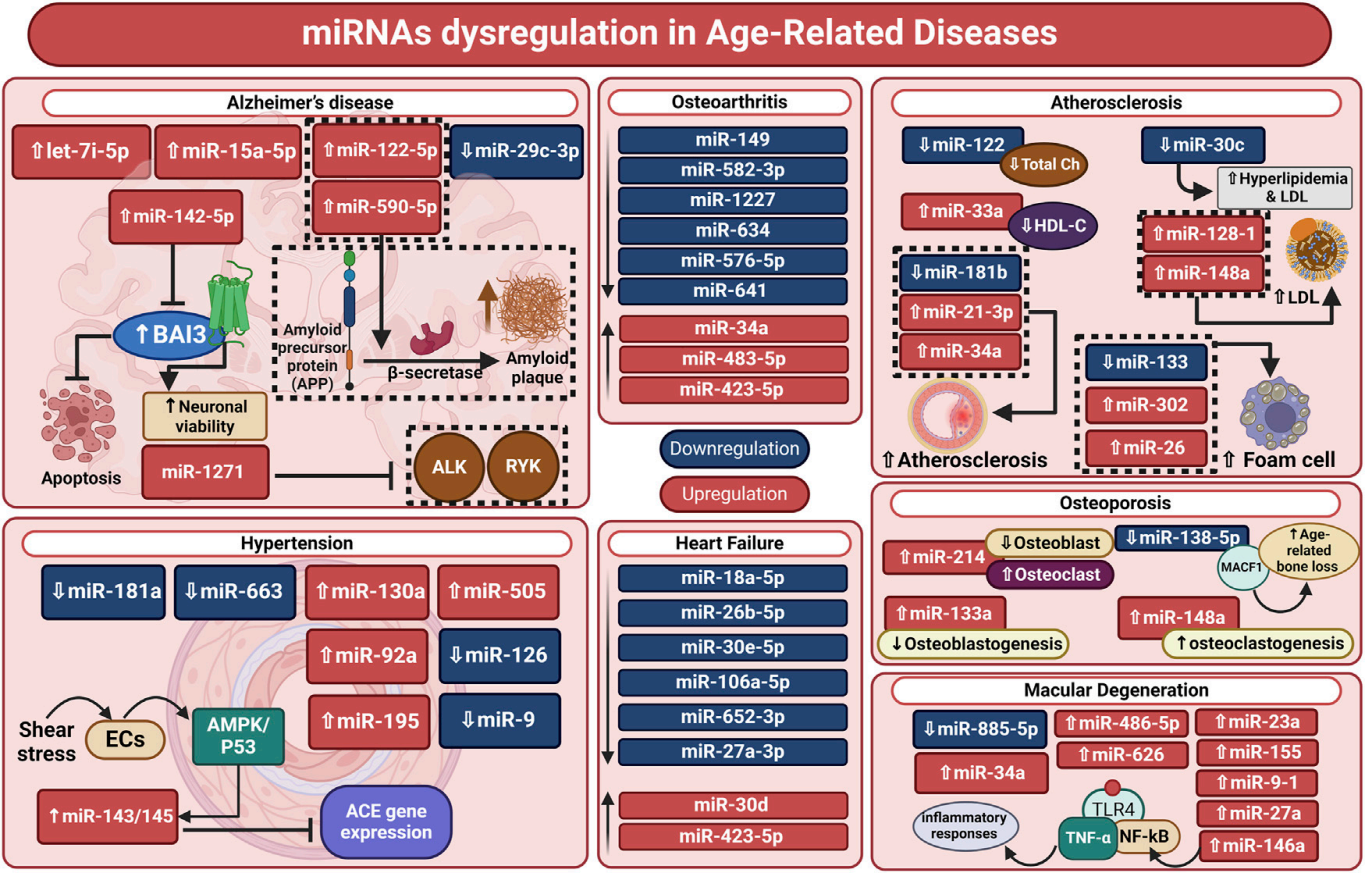
miRNA dysregulation in age-related diseases. Neurodegenerative and age-related disorders, including Alzheimer’s disease, cardiovascular conditions (hypertension, heart failure, and atherosclerosis), osteoarthritis, osteoporosis, and macular degeneration, are strongly associated with miRNA dysregulation. MicroRNAs are involved in disease etiology and may be biomarkers and therapeutic targets. Furthermore, miRNA dysregulation is crucial to understanding age-related diseases’ biological processes as well as personalized therapy. ACE: angiotensin-converting enzyme; ALK, anaplastic lymphoma kinase; AMPKs: AMP-activated protein kinases; BAI3, brain angiogenesis Inhibitor-3; ch, cholesterol; ECs, endothelial cells; HDL-C, high-density lipoprotein cholesterol; LDL, low-density lipoprotein; MACF1, Microtubule Actin Crosslinking Factor 1; miRNA, microRNA; NF-κB: nuclear factor kappa beta; RYK, receptor-like tyrosine kinase; TLR4: Toll-like receptor 4.

Through the G protein-coupled receptor BAI3, miR-142-5p stimulates neuronal synaptotoxicity and prevents apoptosis; its downregulation in the brain of an AD animal model may increase BAI3 expression. In cultured neurons, inhibition of miR-142-5p restores spatial learning and memory (Fu et al., 2021). Furthermore, Blood samples from AD patients show dysregulation of miR-122-5p, miR-210-3p, and miR-590-5p expression in comparison to healthy controls. Moreover, plasma levels of miR-342-5p may be able to predict the rate of cognitive deterioration in AD (Dakterzada et al., 2021; Mankhong et al., 2022). These results imply that dysregulated miRNAs may be a reflection of neuropathological outcomes in AD patients (Figure 2).

Insulin resistance is a major risk factor for Type 2 Diabetes and AD, two of the biggest global health concerns in aging, which have a similar reaction to the regulation of miR-1271 (Arnold et al., 2018). Anaplastic lymphoma kinase (ALK) and Receptor-Like Tyrosine Kinase (RYK), two non-canonical receptor tyrosine kinases (RTKs), have been shown to target miR-1271 in post-mortem AD and Type 2 diabetes tissues. miR-1271 caused the downregulation of both ALK and RYK, which were found to be in charge of the cytoskeleton structural degeneration in both AD and Type 2 diabetes mice (Majumder et al., 2021).

Moreover, exosomes derived from serum and cerebrospinal fluid can be used for miRNA profiling in neurodegenerative diseases such as AD. Many forms of dementia can be distinguished by the degree of expression of miR-193b, miR-135a, and miR-384 in serum and exosomes (Ting et al., 2018; Liu et al., 2021). Furthermore, the ongoing association seen between dysregulated miRNAs, namely miR-132 and miR-212, and cognitive decline in AD patients could lead to advancements in the clinical management of such individuals (Cha et al., 2019).

#### 3.2.2 miRNAs in cardiovascular diseases

Elderly people are more likely to experience cardiovascular troubles due to their aging and fragility, even with advancements in detection and treatment. Over time, age-associated cardiovascular diseases (CVDs) such as atherosclerosis, heart failure, and hypertension can lead to a reduction in an individual’s standard of life and capacity to carry out routine tasks. Important modulators of many biological processes, miRNAs offer an intriguing prospective treatment target for CVDs. Furthermore, identifying trustworthy biomarkers for diagnosis, prognosis, and treatment response prediction is a significant challenge in the field of geriatric medicine. Because of their distinctive hallmark in CVDs and longevity in blood circulation, miRNAs are a very promising approach (Figure 2) (de Lucia et al., 2017).

##### 3.2.2.1 miRNAs in hypertension

One of the most frequent diseases and a major risk factor for renal failure and cardiovascular illnesses is hypertension. Presently, only half of people with hypertension respond to treatment. A vital role in the pathophysiology of hypertension is played by circulating miRNAs, which are becoming useful biomarkers in essential hypertension and are inherently associated with every element of the renin-angiotensin-aldosterone system (Klimczak et al., 2017).

It has been demonstrated that miR-181a and miR-663 directly bind renin mRNA, and their levels were markedly decreased in hypertension individuals (Marques et al., 2011). The miR-143/145 cluster targets the angiotensin-converting enzyme, and it was activated by shear stress through the AMPK-p53 pathway, which decreased the expression of the ACE gene (Kohlstedt et al., 2013). Furthermore, loss of miR-143/145 resulted in overexpression of the Angiotensin-converting enzyme and a change in the phenotype of vascular smooth muscles from contractile to synthetic, which maintains the vascular remodeling that occurs during hypertension and raises the risk of developing neointimal lesions (Boettger et al., 2009). Two investigations additionally demonstrated that miR-21 and mir-221/222 are regulators of aberrant proliferation of vascular smooth muscles and neointimal hyperplasia in rats using a model of balloon-induced vascular wall injury (Chistiakov et al., 2015; Zhang et al., 2016).

Through a comparative analysis of the miRNA expression levels in hypertensive and control patients, it was shown that the human cytomegalovirus-encoded miRNA hcmv-miR-UL112 was upregulated in the diseased patients. This suggests this miRNA may have a role in controlling blood pressure (Li et al., 2011). Using a similar methodology, Yang et al. discovered elevated levels of miR-505 in hypertension after screening plasma samples from three separate patient cohorts (Yang Q. et al., 2014). Another investigation was carried out to find the miRNA signature in exosomes and peripheral blood taken from individuals who showed metabolic risk of CVDs. MiR-130a and −195 correlate positively with blood pressure levels, and are upregulated in hypertension. Likewise, miR-92 was upregulated in cardiovascular and metabolic disorders (Karolina et al., 2012). Furthermore, a study comparing hypertension patients to healthy controls revealed reduced expression levels of miR-9 and miR-126 (Figure 2) (Kontaraki et al., 2014).

##### 3.2.2.2 miRNAs in heart failure

Circulating miRNAs of a cardiac origin mirror alterations in miRNAs seen in cardiac tissue as people age (Vegter et al., 2016). When compared to healthy controls who were at a higher risk of dying, miR-18a-5p, −26b-5p, −27a-3p, −30e-5p, −106a-5p, −199a-3p, −652-3p, and −199a-3p were considerably lower in older individuals suffering acute heart failure (Ovchinnikova et al., 2016). Circulating miR-21, miR-214, and miR-27b were found to be substantially upregulated in ischemia patients, but 13 different miRNAs were downregulated, according to metanalysis done in individuals with heart failure (Gholaminejad et al., 2021). Furtherly, Qiang et al. discovered that miR-126 and −508-5p could be used to predict death in individuals with heart failure (Qiang et al., 2013).

According to a separate investigation, individuals with heart failure-related dyspnea had considerably higher levels of miR-423-5p (Ellis et al., 2013). Based on a translational pilot investigation, miR-30d expression was upregulated as a defense mechanism in cardiac desynchrony zones. Additionally, there is a correlation between the responsiveness to resynchronization therapy and the initial plasma concentration of this miRNA (Melman et al., 2015). Upregulated miR-155 was linked to a favorable outcome from left ventricular assist device implantation, an essential therapy for those suffering from end-stage heart failure (Figure 2) (Wang T. et al., 2017).

##### 3.2.2.3 miRNAs in atherosclerosis

Atherosclerosis is a multi-stage inflammatory disease of the arterial wall that is distinguished by endothelial dysfunction and lipoprotein accumulation, causing gradual remodeling of the artery intima. As the lesion ages, it becomes more advanced and eventually leads to the development of atherosclerotic plaque, which obstructs blood vessels and causes thrombotic episodes. Recent research has yielded new molecular insights into the regulation and clinical diagnosis of atherosclerosis and the corresponding miRNAs in aging related manner (Sharma et al., 2022).

Lipid profile dysregulation is regarded as a primary risk factor for atherosclerosis. miRNAs that can control the production of LDL and HDL reveal a potential therapeutic target for this disease. According to *in vivo* research, mice and non-human primates with miR-122 suppression had lower plasma levels of total cholesterol (Huang et al., 2022). In mice fed a high-fat diet, hepatic upregulation of miR-30c reduced hyperlipidemia and LDL production, and lessened lesions of atherosclerosis, while miR-33a decreased HDL-C in postprandial lipemia (Yaman et al., 2021). Numerous miRNAs were also shown to be connected with dyslipidemia when they were located close to loci for single-nucleotide polymorphisms in a genome-wide association investigation. Among these, the expression of many proteins involved in lipid metabolism and trafficking is regulated by miR-128-1, miR-148a, miR-130b, and miR-301b (Figure 2) (Wagschal et al., 2015).

MiR-148a and miR-128-1 inhibition increased LDL clearance in C57BL/6J mice and altered the amounts of lipoproteins in circulation in ApoE^−/−^ mice given a western diet (Santos et al., 2021). Furthermore, in both mice and monkeys, suppression of miR-33 raised the amounts of HDL cholesterol in the bloodstream. Anti-miRNA-treated animals displayed decreased atherosclerotic lesion size and were able to infiltrate plaque macrophages and reduce the expression of inflammatory genes (Rayner et al., 2011; Goedeke et al., 2014).

The fact that atherosclerosis is a chronic inflammatory condition was the focus of numerous investigations. Data has demonstrated that miR-181b is a key modulator of inflammation in the endothelium. MiR-181b decline might contribute to the formation of atherosclerosis, as excessive expression of this miRNA managed to hinder leukocyte recruitment at the atherosclerotic plaque both *in vitro*, as well as in the plasma of patients suffering from coronary artery disease (Sun et al., 2014). By using a nanoparticle approach, Ma et al., 2016 have devised a procedure to deliver miR-146a and miR-181b directly to the atherogenic lesion, allowing them to decrease the size of atherosclerotic plaque and downregulate the production of chemokines. Through targeting genes involved in cholesterol metabolism, miR-27a/b also regulates the homeostasis of macrophages within the plaque (Zhang et al., 2014). A recent study demonstrated the pivotal function that miR-34a plays in controlling inflammation, atherosclerosis, and macrophage cholesterol export, in which reversing diet-induced metabolic problems and promoting atherosclerosis regression are achieved with therapeutic suppression of miR-34a (Xu et al., 2020).

MiR-133 has been found by Gabunia et al., 2017 to be a regulator of the production of foam cells in vascular smooth muscle, which lowers the proliferation and uptake of oxidized LDL by these cells. As a result, miR-133 might be a useful target for treating inflammatory vascular disorders. Further, it has been demonstrated that other miRNAs, such as miR-302 and miR-26, promote the development of foam cells (Johnson, 2019). Additionally, by promoting vascular smooth muscle migration and proliferation, exosomal miR-21-3p from nicotine-treated macrophages may hasten the onset of atherosclerosis (Zhu et al., 2019).

Additionally, miR-33 was discovered to be an essential regulator of lipoprotein metabolism and cellular lipid homeostasis. It also controls downstream target genes, such as ATP-binding cassette transporter G1 (ABCA1 and ABCG1). The preventive effects of miR-33 loss on the progression of atherosclerosis are due to its actions on macrophages. Through the activation of ABCA1 and ABCG1 in macrophages, therapeutic suppression of miR-33 in mice and nonhuman primates increases HDL levels and prevents the advancement of atherosclerosis by either boosting HDL or improving cholesterol outflow (Zhang et al., 2022). Furthermore, there are variations in the distribution and control of the miR-33 family, especially the advancement of atherosclerosis; miR-33b is thought to be more effective than miR-33a. Indeed, the mice deficient in apolipoprotein E/miR-33a^−/−^/miR-33b^+/+^ developed higher levels of atherosclerotic plaque when fed a diet high in fat and cholesterol than the mice deficient in apolipoprotein E/miR-33a^+/+^/miR-33b^−/−^, which was consistent with the liver’s prevalent levels of miR-33b and a deteriorated lipid profile (Koyama et al., 2019).

Early detection of susceptible carotid plaques may be useful in identifying high-risk stroke patients who could benefit from revascularization sooner rather than later. Atherosclerotic plaque progress biomarker miR-200c might be clinically helpful in identifying patients who are at high risk of embolism (Magenta et al., 2018). Endothelial dysfunction is more likely to occur in situations when oxidative stress is elevated, such as ischemia and aging. Recently, the relationship between miR-200c and sirtuin 1 (SIRT1), endothelial nitric oxide synthase (eNOS), and forkhead box O1 (FOXO1), three closely related proteins that regulate endothelial cell function and ROS production was examined. Through a decrease in NO and an increase in the acetylation of SIRT1 targets, FOXO1, and p53, miR-200c directly targets these proteins. Acetylation of FOXO1 reduced its transcriptional impact on target genes, namely, SIRT1, catalase, manganese superoxide dismutase, and ROS scavengers. Consequently, miR-200c strengthened this molecular circuitry by upregulating ROS and suppressing FOXO1 transcription. Furthermore, anti-miR-200c therapy restored limb perfusion and saved these targets in the mouse model of hindlimb ischemia (Carlomosti et al., 2017).

Circulatory miRNAs are now known as a novel class of atherosclerosis biomarkers, which surfaced to help with clinical diagnosis and open up new treatment options. A study was performed on candidate tissue-derived miRNAs from atherosclerotic plaque in individuals with stable and unstable coronary artery disease. MiR-125b-5p and miR-193b-3p were boosted in individuals with stable coronary artery disease, while miR-223-3p and miR-142-3p were upsurged in those with unstable type, suggesting that these candidate tissue-derived miRNAs could be markers of plaque instability (Singh et al., 2020).

#### 3.2.3 miRNAs in bone and cartilage age-related conditions

Based on the available research, it is clear that miRNAs are strongly involved in the regulation of musculoskeletal system disorders and age-related events. Chiefly in frailty, miRNAs have been found to have an impact on the start and progression of age-related musculoskeletal diseases, including osteoporosis and osteoarthritis (Castanheira et al., 2021).

##### 3.2.3.1 miRNAs in osteoarthritis

Numerous investigations have exhibited the significance of miRNAs in the process of cartilage formation, preservation, and degradation (Vonk et al., 2014). Using chondrocytes derived from osteoarthritis patients, the cells of donors with osteoarthritis showed higher levels of miR-483-5p. On the other hand, through affecting pathways linked to cartilage function, such as SIRT1, miR-149, −582-3p, −1227, −634, -576-5p, and −641 were downregulated in osteoarthritis (Díaz-Prado et al., 2012). Since suppressing miR-34a in an osteoarthritis rat model reduced cartilage breakdown, elevated levels of miR-34a are thought to contribute to osteoarthritis (Tao et al., 2020). Overexpression of miR-146a was also found to have a beneficial effect on cartilage preservation in an osteoarthritis animal model, while its downregulation hinders apoptosis and augments autophagy of chondrocytes (Zhang et al., 2021).

A meta-analysis was conducted recently to identify miRNAs that exhibit abnormal expression as osteoarthritis progresses in the elderly. According to the study, miR-146a-5p and miR-34a-5p were the most elevated miRNAs, while miR-127-5p and miR-140-5p were the most suppressed miRNAs. In osteoarthritis, mesenchymal stem cells and transforming growth factor-β were discovered to be the primary downstream effectors regulated by these miRNAs (Liu et al., 2023).

An efficient drug delivery method was recently created to improve miR-124 stability and its ability to reach chondrocytes, which is thought to be a strong target for its anti-inflammatory properties. By reducing chondrocyte apoptotic cascade, repressing extracellular matrix breakdown, and improving synovial thickness, the tetrahedral framework nucleic acids launching miR-124 efficiently prevent osteoarthritis from progressing and effectively preserve articular cartilage (Shi et al., 2023).

In another recent investigation on cartilage aging and osteoarthritis, multiple miRNAs were found to be differently expressed in youthful intact compared to osteoarthritic lesioned cartilage. After IL-1β treatment, the expression of miR-107, miR-143-3p, miR-361-5p, and miR-379-5p were declined in human primary chondrocytes. Additionally, the study demonstrates the significance of miR-107 and miR-143-3p in controlling protein translation, proliferation, and hypertrophy in chondrocytes (Figure 2) (Balaskas et al., 2023).

##### 3.2.3.2 miRNAs in osteoporosis

It has also been demonstrated that miRNAs have a significant role in other age-related illnesses like osteoporosis and osteopenia. For instance, miR-133a inhibits osteoblastogenesis and may be a possible biomarker because osteoporosis patients have greater levels of miR-133a than do postmenopausal women in good health (Li Z. et al., 2018). Recently, miR-214 has been considered a potential marker of osteoporosis as its expression has a negative correlation with recognized markers of bone production (Sadu et al., 2023). Furthermore, in the femoral condyles of osteoporotic rats, anti-miRNA-214 increased osteoblast activity and decreased osteoclast activity, enhancing bone metabolism and delaying the onset of osteoporosis (Wang et al., 2023). It has been shown that miR-138-5p targets microtubule actin cross-linking factor 1 to control the differentiation of aged osteoblasts. Ultimately, the reduction in bone production and age-related bone loss in elderly mice was compensated by the therapeutic suppression of miR-138-5p (Chen et al., 2022). Additionally, by removing Forkhead box O1, miR-182 inhibits the osteogenesis of bone-forming osteoblasts (Chen et al., 2019). However, decreasing miR-148a expression *in vivo* resulted in reduced osteoclast formation and elevated bone mass in ovariectomized and control animals. Meanwhile, MiR-148a was demonstrated to promote osteoclastogenesis *in vitro* (Figure 2) (Tian et al., 2021; Pan et al., 2022).

#### 3.2.4 miRNAs in macular degeneration

One of the leading causes of blindness in the world is age-related macular degeneration or AMD. Recent research indicates that epigenetic mechanisms, such as the regulation of gene expression by miRNAs, may be significant to AMD in addition to environmental and genetic variables, offering a promising new direction for therapy and research (Berber et al., 2017). According to a recent investigation, miR-885-5p was considerably downregulated in the serum of AMD patients, while miR-486-5p and miR-626 were more expressed than in the control group. The apoptotic and neovascularization pathways, which are involved in the pathophysiology of AMD, are known to depend critically on these miRNAs (Elbay et al., 2019). Other involved pathways in the pathophysiology of AMD include amyloid-β retinal deposition, which triggers apoptotic and inflammatory responses in addition to varying miRNA expression, thus causing dysregulation of the transforming growth factor-β pathway. Furthermore, it has been demonstrated that miR-155 and miR-27a impact 42 genes implicated in the transforming growth factor-β route, while miR-146a can target genes involved in inflammatory pathways, including nuclear factor-κB, tumor necrosis factor signaling pathways, and Toll-like receptors (Romano et al., 2017).

Additionally, IL-2, STAT3, and ERK were found to be a novel global pathway with activation hallmarks of AMD that are implicated in the cell-based inflammatory response (Makarev et al., 2014). Four miRNAs (miRNA-9, miRNA-125b, miRNA-146a, and miRNA-155) were shown to be increased in AD and AMD, according to the most recent research. The expression of complement factor H, a key inhibitor of the innate immunological and inflammatory response that is implicated in both AMD and AD, is subsequently downregulated because of these increased miRNAs. It has been shown that there are many increased miRNAs in the retina of AMD patients, which are also common in complement factor H deficiency, resulting in inflammatory neurodegeneration. MiRNA-7, miRNA-9-1, miRNA-23a/miRNA-27a, miRNA-34a, miRNA-125b-1, miRNA-146a, and miRNA-155 are highly expressed in the retinal macular region afflicted by AMD and the AD-affected superior temporal lobe neocortex (Figure 2) (Hill et al., 2015; Pogue and Lukiw, 2018).

Treatment targets for AMD are thought to include several miRNAs implicated in either retinal pigment epithelium atrophy or choroidal neovascularization (Urbańska et al., 2022). For neovascular AMD, anti-vascular endothelial growth factor therapy is a crucial treatment (Cornel et al., 2015). It has been established that a certain subset of miRNAs is crucial to angiogenesis. The miR-15/107 group, the miR-17∼92 cluster, miR-21, miR-132, miR-296, miR-378, and miR-519c are some of these miRNAs. It is thought that miR-23a mimics reduce macular related-apoptosis whereas miR-23a inhibition increases it (Ren et al., 2017; Urbańska et al., 2022).

The pathogenic significance of the newly detected miR-19a, miR-126, and miR-410 in AMD was determined by another recent bioinformatics technique. There was a substantial link found between AMD pathogenesis and these miRNAs, and their target genes. Because of this, they might serve as promising new targets for AMD patients’ treatments or prognostic biomarkers (ElShelmani et al., 2020). Using exosomes produced from AMD, the study also explained the functional roles of miRNAs in in vitro human cell line models. The findings show that, when compared to the control group, the expression of human apoptotic miRNAs is more impacted by dry AMD-derived exosomes than by wet-derived ones. After being exposed to dry AMD-derived exosomes, the used cell line expressed greater levels of let-7a-5p, miRNA-17-5p, miRNA195-5p, miRNA26b-5p, and miRNA-30c-5p (ElShelmani et al., 2021b).

### 3.3 Potential implication of miRNA in clinical trials to enhance longevity

With the first FDA-approved small RNA drugs entering clinical medicine, continuous research for the microRNA (miRNA) class of small RNAs has expanded its preclinical and clinical research application. The growing evidence from the huge number of reports indicates that miRNAs could be put to significant utility as biomarkers for pathogenic conditions, modulators of drug resistance, and/or drugs for medical intervention in almost all human health conditions (Hanna et al., 2019). In a study involving 16 non-Hispanic men aged between 50 and 60, whose lifespans ranged from 58 to 92 years, PCR arrays were used to track changes in miRNA levels in their serum over time. Notably, variations in the expression of these miRNAs were observed. Specifically, at the age of 50, 24 miRNAs were found to be significantly more active, while 73 showed reduced activity in those who lived longer (76–92 years), compared to those who had shorter lifespans (58–75 years). For the group that lived longer, miR-373-5p showed the highest increase in activity, whereas miR-15b-5p was the most decreased. Over the years, a strong correlation between the lifespan and the activity levels of nine specific miRNAs was observed, with significant changes noted in six of these (miR-211-5p, 374a-5p, 340-3p, 376c-3p, 5095, 1225-3p) when comparing the longer-lived to the shorter-lived participants. These six miRNAs affect 24 proteins linked to aging, such as PARP1, insulin-like growth factor-1 receptor (IGF1R), and IGF2R. Based on the findings, activity patterns of these six miRNAs are suggested to be valuable indicators of aging (Smith-Vikos et al., 2016). This suggests that a single miRNA candidate could have the ability to control whole biological pathways related to aging, thereby promoting longevity.

Despite significant advancements in preclinical studies, miRNA-based therapies are still in the preliminary phase of development. A limited number have advanced to clinical trials, with none making it to phase III or receiving approval from the FDA. Additionally, some have been discontinued due to concerns about toxicity. These obstacles underscore the need to overcome current obstacles to fully realize the potential of miRNA-based treatments in clinical settings (Seyhan, 2024). Currently, no miRNAs are undergoing clinical trials for longevity enhancement. However, the promise of miRNAs as treatments for a range of diseases is evident, and further research is crucial to assess their practicality in clinical environments.

## 4 Impact of nutritional factors and nutraceuticals on miRNA modulation

This review will discuss recent studies investigating the impact of nutritional factors and nutraceuticals on aging, which affect miRNA functions or expression. The interrelation between nutrition and age-related diseases has been studied for years as malnutrition, cachexia, or weight loss are related to cancer or other diseases (Witwer, 2012).

### 4.1 Impact of macronutrients on miRNA expression and function in aging

Macromolecules such as carbohydrates, fatty acids, and amino acids can protect or induce aging through miRNA modulation. Beta cell dysfunction is associated with overexpressed miR-29a in beta cells of Langerhans supplemented by high glucose media (Bagge et al., 2012). Moreover, miR-29a is also induced by saturated fatty acids repressing insulin receptor signaling −1 (IRS-1) and increasing insulin resistance in myocytes (Yang et al., 2014c) (Table 2). A high-fat diet is shown to increase the expression of miR-21 which promotes liver carcinogenesis (Heng et al., 2016). In addition, a high-fat diet could overexpress miR-195 impairing insulin sensitivity and glycogen metabolism in the liver (Yang et al., 2014d). Moreover, a high-fat diet is involved in the pathogenesis of atherosclerosis through upregulation of miR-210 inducing endothelial cell apoptosis (Li et al., 2017).

**TABLE 2:** Role of nutraceuticals and nutrition in miRNA regulation in aging.

Nutrient or nutraceuticals	Nature	Affected miRNAs	Expression change	Impact	Ref.
Glucose	Macronutrient	miR-29a	Upregulated	Beta cell dysfunction	(Bagge et al., 2012)
	Macronutrient	miR-21	Upregulated	Mediate the nephropathy complication	(Dey et al., 2011)
Saturated fatty acids	Macronutrient	miR-29a	Upregulated	Increase insulin resistance in myocytes	(Yang et al., 2014c)
Fat	Macronutrient	miR-21	Upregulated	Increased lipid accumulation in the liver and carcinogenesis	(Heng et al., 2016)
	Macronutrient	miR-195	Upregulated	Impairs insulin sensitivity	(Yang et al., 2014d)
	Macronutrient	miR-210	Upregulated	Induce endothelial cell apoptosis and atherosclerosis	(Li et al., 2017)
Protein	Macronutrient	miR-200, miR-192	Downregulated	Decrease epithelial to mesenchymal kidney transition	(Sene et al., 2018)
Xylobiose	Macronutrient	miR-122a/33a	Upregulated	Reduce hepatic lipogenesis in diabetes mellitus	(Lim et al., 2016)
Selenium	Micronutrient	miR-185	Upregulated	Increase expression of selenoproteins such as GPX2	(Maciel-Dominguez et al., 2013)
	Micronutrient	miR-374	Downregulated	Cardiac function	(Xing et al., 2015)
Selenium and resveratrol	Micronutrient	miR-134	Downregulated	Alleviate neuroinflammation in Alzheimer’s disease	(Abozaid et al., 2022)
Vitamin D	Micronutrient	miR-148a and miR-122-5p	Downregulated	Improve bone health	Al-Rawaf et al. (2021)
Zinc	Micronutrient	miR-223, miR-21, and miR-31	Downregulated	Reduce esophageal cancer risk	Fong et al. (2016)
Vitamin B12 Folic acids	Micronutrients	miR-483	Downregulated	Protect against type 2 diabetes mellitus in the offspring’s adult life	(Mahajan et al., 2019)
Olive oil	Rich in phenolics	miR-484, miR-137-3p, miR-27, and miR-124-3p	Upregulated	Reduce brain plasticity and improves neurological and behavioral function	(Luceri et al., 2017a)
Grape seed	Flavonoid, procyanidin	miR-1249, miR-483, miR-30c-1, and miR-3544	Altered expression	Improve pancreatic function in diabetes	(Castell-Auví et al., 2013)
Pistachio	Others	miR-192 and miR-375	Downregulated	Reduce plasma glucose level and improve insulin resistance	(Hernández-Alonso et al., 2017)
Probiotics	Others	miR-181a and miR-155	Downregulated	Modulate the inflammatory response of systemic lupus erythematosus	(Vahidi et al., 2018)
	Others	miR-34	Downregulated	Enhance health and wellbeing	(Haslberger et al., 2021)
Nicotinamide mononucleotide	Others	miR-146a, and miR-203	Upregulated	Improve the health of the aged aorta	(Kiss et al., 2019)
	Others	miR-99b and miR-127	Downregulated		

Hyperglycemia also induces the expression of miR-21 reducing the tumor suppressor protein phosphatase and tensin homolog deleted in chromosome 10 (PTEN) mediating the pathogenesis of nephropathy in diabetic patients (Dey et al., 2011). While protein is important for kidney health, low maternal protein intake accelerates epithelial-mesenchymal transition in the later adult life of the offspring through increasing expression of miR-192 and miR-200 (Sene et al., 2018). Xylobiose intake as an alternative to sucrose and present in bamboo can be favorable for diabetic patients as it regulates hepatic lipogenesis by overexpressing miR-122a/33a resulting in reducing triglyceride levels and inflammatory cytokines (Lim et al., 2016).

### 4.2 Influence of micronutrients and trace minerals on miRNA regulation

Non-optimum levels of micronutrients such as selenium, zinc, and vitamin D generate several aging-related diseases (McCann and Ames, 2011; Prasad, 2012; Sinha et al., 2013). They are included in miRNA modulation being included in their structure. Selenium is one of the micronutrients that has gained attention recently, due to its incorporation into selenoproteins such as glutathione peroxidase or other antioxidant enzymes (Bellinger et al., 2009). An *in vitro* experiment utilizing colorectal cancer cell lines in a selenium-deficient environment results in a changed expression of miR-185 with a feedback mechanism regulating selenoproteins as glutathione peroxidase 2 (Maciel-Dominguez et al., 2013). In selenium-deficient rats, miR-374 is overexpressed leading to a dysregulated Wnt signaling pathway and cardiac dysfunction (Xing et al., 2015). Moreover, combining resveratrol with selenium nanoparticles enhanced the effectiveness of resveratrol against Alzheimer’s disease reducing neuroinflammation through downregulating miR-134 (Abozaid et al., 2022). Vitamin D deficiency in diet upregulates miR-148a and miR-122-5p and it is related to bone loss and eventually osteoporosis in post-menopausal women (Al-Rawaf et al., 2021). Moreover, zinc deficiency is linked to an increased risk of esophageal cancer due to overexpression of miR-223, miR-21, and miR-31 (Fong et al., 2016).

**FIGURE 3:**
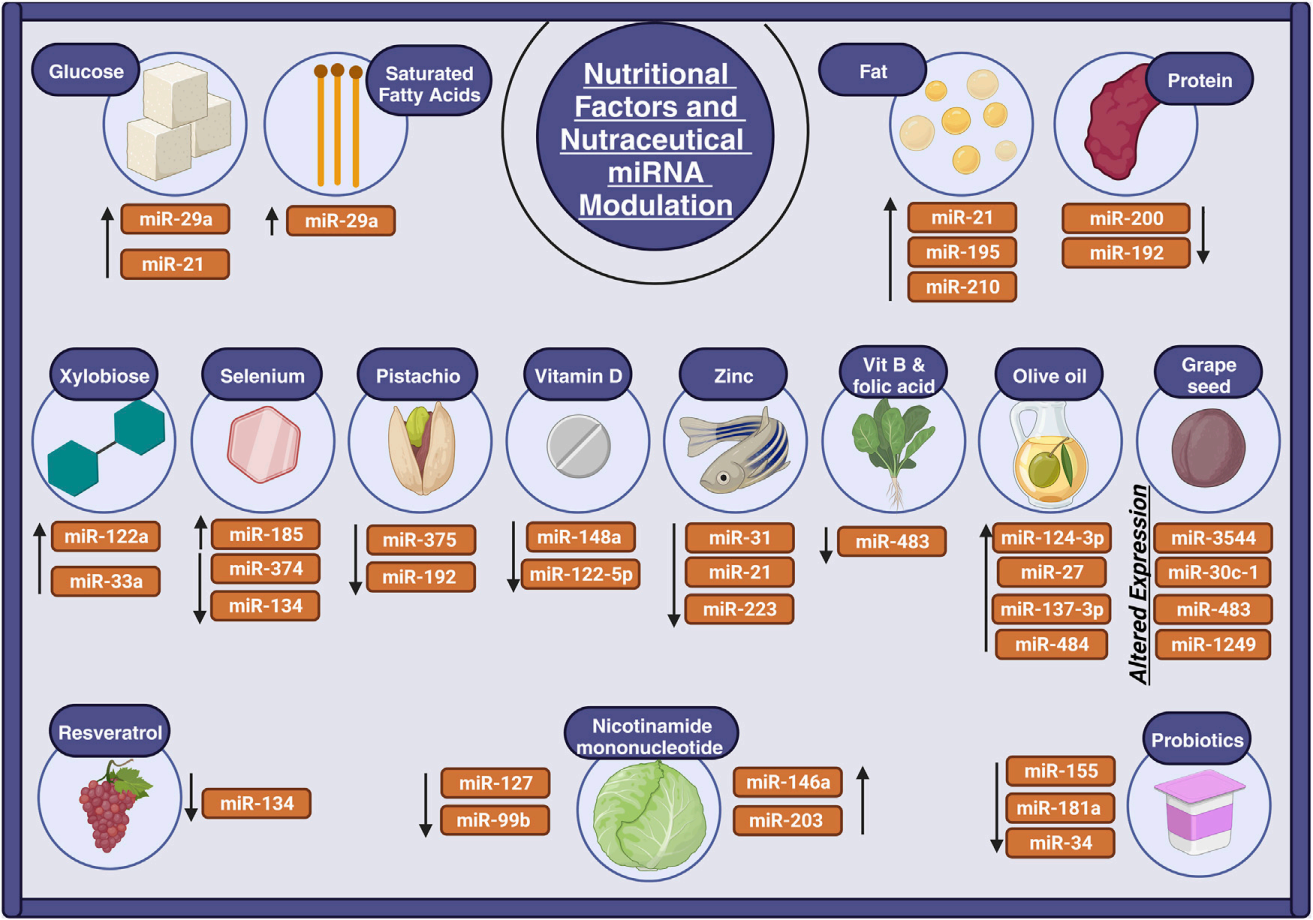
Nutritional factors and nutraceuticals’ role in miRNA regulation. The illustration depicts how nutrients and nutraceuticals modulate miRNAs in aging processes. MiRNA expression is influenced by macronutrients such as carbohydrates and fatty acids, as well as micronutrients like selenium and zinc, which impact aging and age-related diseases. Flavonoids and polyphenols in olive oil and grape seeds influence miRNA function in the fight against aging. Nutraceuticals, such as pistachios and probiotics, also affect miRNA expression, suggesting promising treatments for age-related diseases.

Vitamin B12 and folic acid are essential for several biological processes and are also important for maternal nutrition for the offspring’s health. Imbalanced nutritional levels in maternity are linked to insulin resistance and type 2 diabetes mellitus in the offspring’s adult life through upregulating miR-483 which limits fats in the adipose tissue (Figure 3) (Mahajan et al., 2019).

### 4.3 Modulation of miRNAs by flavonoids and polyphenols

Phenolic compounds or flavonoids act as anti-aging agents by regulating miRNA function. Olive oil rich in phenols intake in middle age increased the miRNA expression of miR-484, miR-137-3p, miR-27, and miR-124-3p resulting in reduced brain plasticity and improving neurological and behavioral function (Luceri et al., 2017a). Another study investigated the impact of grape seed intake rich in procyanidin on diabetic rats, it was found that the flavonoids of grape seed modulate glucose metabolism and improve pancreatic function through downregulating the expression of miR-1249, miR-483, miR-30c-1, and upregulating miR-3544 expression (Figure 3) (Castell-Auví et al., 2013).

### 4.4 Other nutraceuticals and their effects on miRNA expression

Chronic pistachio intake, rich in unsaturated fat, minerals, and vitamins, modulates insulin resistance through downregulating miR-192 and miR-375 involved in increasing plasma glucose levels and insulin resistance (Hernández-Alonso et al., 2017) (Table 2). Probiotics modulate different inflammatory diseases. For instance, probiotics such as *Lactobacillus rhamnosus* and *Lactobacillus delbrueckii* downregulate the expression of miR-181a and miR-155 involved in the inflammatory response of systemic lupus erythematosus (Vahidi et al., 2018). In addition, the intake of probiotics such as bifidobacterium modulates cell cycle senescence by regulating miR-34 and increasing Sirtuins 1 resulting in wellbeing and reduced aging (Haslberger et al., 2021). Nicotinamide mononucleotide supplementation enhances the antiaging properties of the aging aorta by upregulating miR-146a, and miR-203 and downregulating miR-99b and miR-127 (Figure 3) (Kiss et al., 2019).

## 5 Mechanisms and pathways

### 5.1 Molecular mechanisms underlying miRNA-mediated regulation of aging processes

MiRNAs are increasingly acknowledged as key players in aging and longevity. Several miRNAs directly impact lifespan by affecting major aging pathways. While most insights into miRNAs that influence longevity come from invertebrate studies, the mechanisms, and roles of miRNAs in aging are similarly observed in mammals.

Mechanisms in which miRNAs control gene expression often involve their “seed” sequences interacting mostly with the 3′-end, and less frequently with the 5′-end, of mRNA that is transcribed from targeted genes. In the past decade, many studies have focused on both quantitative and qualitative evaluations of miRNA expression, revealing significant alterations in miRNA expression patterns in different diseases. Consequently, analyzing miRNA expression profiles can be a crucial method for diagnosing and treating diseases (Gulyaeva and Kushlinskiy, 2016).

Cellular and molecular damage gradually accumulates over time, triggering the intricate process of aging. Ultimately, this accumulation results in a generalized decline in physiological functions, an elevated risk of mortality, and the end of life. Even while a variety of environmental and random circumstances have a role in an individual’s aging process, there is a significant basic inherited component to aging too. It has been proposed that systems crucial for organism development and cell proliferation may indirectly affect gene expression patterns that govern aging and senescence (Campisi, 2005). Furthermore, it has been found that several elements that play a universal function in all species control how long *Caenorhabditis elegans* survives. These include heat-shock factors (HSFs), sirtuins, AMP-activated protein kinases (AMPKs), mitogen-activated protein kinases (MAPKs), and signaling through the insulin/insulin-like growth factor-1 (IGF-1) signaling (IIS) pathway (Smith-Vikos and Slack, 2012).

#### 5.1.1 Interplay between insulin signaling and the aging process

The most studied system in aging research, the IIS Signaling pathway, was initially found to control longevity in *C. elegans* (Friedman and Johnson, 1988; Kenyon et al., 1993). The insulin or IGF-1 receptor, also known as DAF-2 in *C. elegans*, is activated in this pathway. This activation starts a series of subsequent events that eventually control longevity. In situations of gathering and resource scarcity during development, DAF-2 affects the organism’s decision to enter dauer, a dormant state. In adulthood, DAF-2 reacts to insulin-like peptides that are mostly released by neurons in maturity, when its function changes to controlling longevity on a cellular level. Through a phosphorylation cascade involving PI3K (AGE-1 in *C. elegans*), the phosphatidylinositol (PtdIns)-dependent kinase (PDK) (counteracted by DAF-18, the *C. elegans* counterpart of the PtdInsP3 phosphatase PTEN), AKT, and SGK, it suppresses the FOXO transcription factor, DAF-16 in *C. elegans*. DAF-16 travels to the nucleus while this sequence is inactive, but phosphorylation retains it in the cytoplasm. It either activates or inactivates several genes that control metabolism, pathogen resistance, heat-shock proteins, superoxide dismutase, catalase, and metallothionein, among other components of the cellular stress response. This coordinated reaction results in increased longevity (Zhu et al., 2010).

**FIGURE 4:**
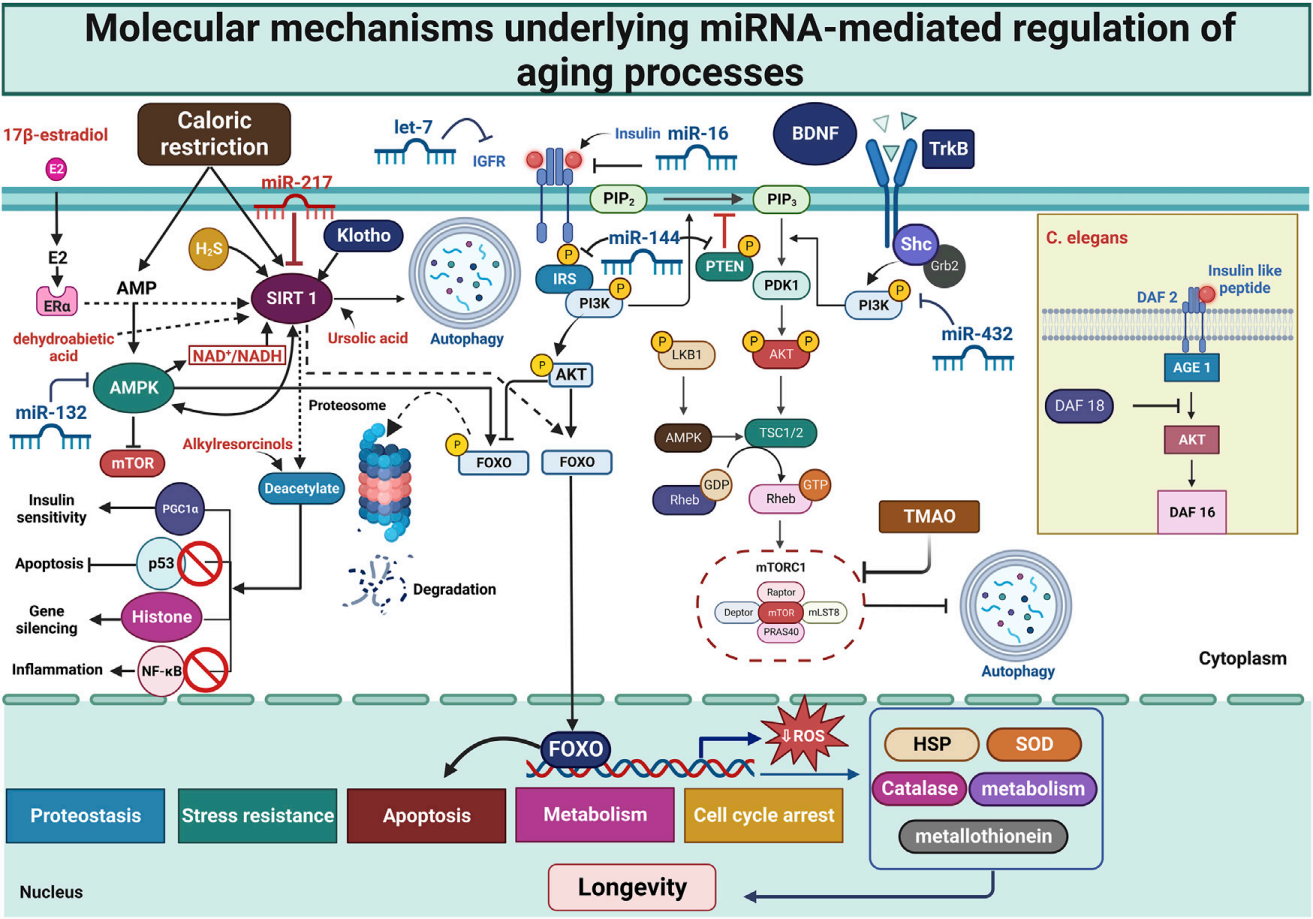
Molecular mechanisms underlying miRNA-mediated regulation of aging processes. This figure illustrates the molecular basis of miRNA-mediated control over aging, with a focus on nutrition-sensing pathways and aging-related signaling networks. Across various species, miRNAs play a crucial role in regulating lifespan and key aging mechanisms. Lifespan and age-related processes are governed by AMPK, Sirtuins, and the Insulin/IGF-1 signaling pathways. The interconnection between cell metabolism, stress response, and longevity is established through nutrient-sensing mechanisms such as mTOR and the IGF1/PI3K/AKT pathway. Diet affects miRNA modulation, highlighting the complex link between nutrition, molecular aging pathways, and lifespan regulation. A comprehensive understanding of the complex interplay between miRNAs and nutrient-sensing pathways can shed light on the causes of aging and identify potential drug targets. AGE, advanced glycation end products; Akt, protein kinase B; AMP, adenosine monophosphate; AMPKs, AMP-activated protein kinases; BDNF, brain-derived neurotrophic factor; ER, estrogen receptor; FOXO, Forkhead box protein O; HSP, heat shock protein; IGF-1, insulin-like growth factor-1; IGFR, insulin-like growth factor receptor; IRS, insulin receptor substrate; mTOR, mammalian target of rapamycin; mTORC, mTOR complex; NF-κB, nuclear factor kappa beta; PDK1, 3-phosphoinositide-dependent kinase 1; PGC1α, peroxisome proliferator-activated receptor gamma coactivator 1-alpha; PI3K, phosphoinositide-3-kinase; PIP2, phosphatidylinositol (4,5)-bisphosphate; PIP3, phosphatidylinositol (3,4,5)-trisphosphate; PRAS40, proline-rich Akt substrate of 40 kDa; PTEN, Phosphatase and Tensin Homolog; ROS, reactive oxygen species; SIRT1, Sirtuin 1; SOD, superoxide dismutase; TGF-β, Transforming growth factor β; TLR4, Toll-like receptor 4; TMAO, trimethylamine N-oxide; TrkB, Tropomyosin receptor kinase B; TSC, Tuberous sclerosis protein.

It has been determined that DAF-2, DAF-16, and HSF-1 are all involved in the same pathway as LIN-4 and LIN-14 (Boehm and Slack, 2005). This suggests a theoretical approach where DAF-2 and LIN-14 either concurrently downregulate DAF-16’s function or where LIN-14 might function upstream of DAF-2. Furthermore, DAF-16 has been shown to decrease lin-4 expression, suggesting that there may be a negative feedback loop between lin-4 and DAF-16. It's crucial to remember that the aging process has not yet been proven to involve this particular mechanism (Figure 4) (Baugh and Sternberg, 2006).

#### 5.1.2 Role of AMP-Activated protein kinases in the aging process

Under regular circumstances, the AMPK, known for its role in prolonging longevity, is primarily responsible for maintaining metabolic balance and autophagy. This involves the elimination of damaged cellular components and molecules. A variety of molecules from the sirtuin family, known for their life-span extension capabilities, are involved in regulating metabolism and repairing DNA damage. These molecules have either mono-ADP-ribosyltransferase or deacylase activity. Additionally, another group of factors promoting longevity includes FOX (forkhead box) proteins. These proteins are a class of transcription factors that regulate the expression of genes linked to the development, division, growth, and lifespan of cells. Nicotinamide mononucleotide (NMN), a precursor to NAD^+^ that is associated with longer life spans, is produced by the enzyme nicotinamide phosphoribosyltransferase (NAMPT) (Siamak, 2020). Through the renewal of autophagic flux and the NAD^+^ synthetic rescue strategy, AMPK protects cells from oxidative stress-induced senescence by raising NAD^+^ levels in aged cells. (Han et al., 2016). The enzyme Klotho, which helps control insulin sensitivity and minimizes oxidative stress, declines with age, accelerating aging in mice. Furthermore, transsulfuration pathways and hydrogen sulfide are essential for preserving cells and extending life. The dysfunction of several cellular pathways, including PI3K/AKT, mTOR, insulin/IGF signaling, and p53, is another hallmark of aging. These pathways can result in immune system deterioration, metabolic problems, and poor cell maintenance. These alterations have the potential to cause cancer by reducing the number of stem cells and inducing senescence or death in cells (Figure 4) (Siamak, 2020).

#### 5.1.3 The impact of SIRT1 signaling on the aging process

Elevated expression levels of sirtuins, especially SIRT2 and SIRT6, have been found to prolong the lifespan in many taxa, including budding yeast (*S. cerevisiae*), nematodes (*C. elegans*), fruit flies (*D. melanogaster*), and mice (Kaeberlein et al., 1999; Tissenbaum and Guarente, 2001; Rogina and Helfand, 2004; Kanfi et al., 2012). Sirtuin affects a variety of biological processes, which helps organisms survive more. More specifically, it has been shown that SIRT1 activation improves insulin sensitivity and lowers insulin resistance (Wang et al., 2019). Alkylresorcinols, belonging to the group of phenolic lipids, stimulate SIRT1-mediated deacetylation. This results in decreased acetylated histone levels in human monocyte cells and contributes to the development of lifespan in *D. melanogaster* (Kayashima et al., 2017). Ursolic acid possesses the ability to activate SIRT1 directly by binding to its external surface. This binding alters SIRT1’s configuration, switching it from a dormant to an active state. This interaction is observed in computational simulations (*in silico*), laboratory experiments *in vitro*, and *in vivo*, and it is significantly important in the aging process (Figure 4) (Kayashima et al., 2017). Kim et al., 2015 have found that dehydroabietic acid, a diterpene resin acid naturally present in conifer trees, can directly activate SIRT1. This activation contributes to reducing the buildup of lipofuscin and decreasing collagen secretion in humans. Additionally, this compound has demonstrated an ability to extend the lifespan of *C. elegans*. A03, a compound developed to interact with ApoE4 and boost SIRT1, has shown effectiveness in enhancing SIRT1 expression in the hippocampus of 5xFAD-ApoE4 (E4FAD) mice, a model for AD. This increase in SIRT1 expression has resulted in improved cognitive abilities in these mice (Campagna et al., 2018). 17β-estradiol triggers the ERα/SIRT1 pathway, which helps reduce oxidative stress, neuroinflammation, and neuronal apoptosis in male mice induced with d-galactose. Additionally, it elevates SIRT1 levels by promoting the degradation of PPARγ through the E3 ubiquitin ligase NEDD4-1, thereby delaying cellular aging (Han et al., 2013; Khan et al., 2019). Despite the numerous positive impacts of SIRT1 on aging and its widespread presence in the body, the mechanisms by which it contributes to anti-aging are not clearly understood.

#### 5.1.4 The influence of mTOR signaling on aging

The fact that mTOR is linked to many aging-related processes, its function as a crucial regulator of longevity and aging has been extensively studied over the last 10 years (Weichhart, 2018; Liu et al., 2019; Papadopoli et al., 2019). While the detailed mechanisms remain partially understood, the link between mTOR signaling and aging is evident across species, from worms to mammals (Vellai et al., 2003; Kapahi et al., 2004; Kaeberlein et al., 2005; Guertin et al., 2006). The Ras-related GTP-binding protein (Rag), which controls amino acid signaling, and the tuberous sclerosis complex (TSC)-Rheb pathway are both involved in the regulation of mTORC1 (Arriola Apelo and Lamming, 2016; Soukas et al., 2019). Numerous studies indicate that mTORC1 responds to various environmental stimuli, including glucose, growth hormones, oxygen, and amino acids. It affects many biological processes, such as autophagy, cell division, and protein synthesis (Widlund et al., 2013; Arriola Apelo and Lamming, 2016). Similarly, mTORC2 is a downstream effector of IIS signaling following PI3K activation and plays a vital role in activating a range of kinases. For example, the important regulator of cell survival, Akt/PKB, is phosphorylated and activated by mTORC2 (Sarbassov et al., 2006; Arriola Apelo and Lamming, 2016). Given that mTOR has negative impacts on aging, it seemed plausible to believe that mTOR expression would increase in the older ages. However, it was found that as people aged, mTOR activity as well as that of its upstream signaling pathways, such as brain-derived neurotrophic factor (BDNF)/PI3K/Akt, decreased (Figure 4) (Yang F. et al., 2014). Numerous negative effects, such as glucose intolerance, diabetes, lowered activity levels, and immunosuppression, have been brought upon by interfering with mTORC2 (Arriola Apelo and Lamming, 2016; Chellappa et al., 2019). As a result, it has been demonstrated that reducing mTORC1 can increase lifespan and slow down the aging process, whereas blocking mTORC2 can have an adverse effect on health and longevity. Age-related deficiencies in spatial learning and memory are reduced when mTOR is long-term suppressed. However, an inverted U-shaped curve characterizing the link between mTOR activity and cognitive function suggests a dose-effect relationship (Chellappa et al., 2019). Trimethylamine-N-oxide (TMAO), an intestinal flora metabolite, suppresses mTOR signaling in SAMP8 and SAMR1 mice, which has a deleterious effect on age-related cognitive decline. This is evidenced by exacerbated synaptic damage and reduced expression of proteins associated with synaptic plasticity (Li D. et al., 2018).

#### 5.1.5 Aging and inflammatory processes

Aging is marked by widespread chronic inflammation, associated with cellular aging, immune system decline, organ malfunction, and various diseases linked to aging. The Senescence-associated secretory phenotype (SASP) refers to a multifaceted process linked with cellular aging, marked by the secretion of pro-inflammatory cytokines from cells that are aging (Olivieri et al., 2015). Essential components of the SASP encompass a range of inflammatory cytokines, chemokines, proteases, and growth factors (Coppe et al., 2008; Davalos et al., 2013; Basisty et al., 2020). This occurrence plays a role in numerous age-related transformations, such as increased inflammation, changes in the immune system, and the proliferation of tumors (Birch and Gil, 2020). Recent research has determined shared components of the SASP across various cell types and triggers of senescence. Some of these components, like serine protease inhibitors, stanniocalcin, and growth differentiation factor 15, are also recognized as markers of aging in human blood plasma (Basisty et al., 2020). Elements such as impaired mitochondria, a persistent DNA damage response, (Rodier et al., 2009), and activation of proteins like C/EBPβ and NF-κB are critical in driving this process (Kulkarni et al., 2020).

#### 5.1.6 Other factors contributing to the aging process

Numerous aging-related biological processes and molecular activities could be associated with ferroptosis. As cells age and undergo alterations in function and metabolism, they may become more prone to external influences, increasing their susceptibility to ferroptosis. Current studies have emphasized the contribution of ferroptosis in the acceleration of aging in skeletal muscles. In this scenario, aging skeletal muscles exhibit reduced expression of Tfr1 and increased expression of Slc39a14, predominantly observed on the cell membranes of skeletal muscle cells in aging mice. The heightened expression of Slc39a14 results in enhanced absorption of non-transferrin-bound iron, causing an accumulation of free iron ions in skeletal muscles, which in turn initiates ferroptosis (Ding et al., 2021).

Additionally, the connection between ferroptosis and aging seems to be bidirectional. Ferroptosis could play a role in triggering various age-related conditions, thereby accelerating the aging process in tissues and cells. On the other hand, the alterations in cellular function and metabolism that occur with aging may increase the cells’ vulnerability to ferroptosis, potentially worsening the progression of diseases.

### 5.2 Signaling pathways involved in miRNA modulation by nutrients and nutraceuticals

Aging research is progressively concentrating on alterations in nutrient sensing pathways, recognizing their potential to be modulated through pharmacological interventions as well as dietary modifications (Longo et al., 2015). Important nutrition sensing mechanisms, including the AMPK/Sirtuin/PGC1 and IGF1/PI3K/AKT/mTOR pathways, can be disrupted by aging. Numerous cellular activities, including protein synthesis, cell cycle progression, DNA replication, autophagy, stress response, and glucose homeostasis, are dependent on these pathways. Environmental factors such as pollution, physical exercise, smoking, and food can alter the biological mechanisms linked to aging.

Caloric restriction (CR) is recognized as an effective dietary strategy to extend lifespan healthily, highlighting diet as a key environmental factor influencing the aging process (Longo et al., 2015; Most et al., 2017). Dietary choices and compounds can impact molecular aging pathways, thereby altering the quality and healthfulness of aging. Recent research indicates that miRNAs play a role in regulating age-related activities, such as DNA replication, cellular aging (senescence), and programmed cell death (apoptosis). Additionally, miRNAs have been found to influence nutrient sensing pathways Thus, miRNAs not only regulate nutrient sensing pathways but can also be influenced by dietary factors (Figure 4) (Micó et al., 2017).

#### 5.2.1 The role of nutrient sensing pathways in molecular aging

Studies indicate that mTOR, the insulin/IGF-1/PI3K/AKT, and the AMPK/SIRT1 pathways are important participants in the process by which CR prolongs life (Johnson et al., 2013; Longo et al., 2015; Altintas et al., 2016; Martins et al., 2016). These pathways demonstrate a connection between nutrition, age, and metabolism. Elevated glucose causes insulin production, which in turn raises IGF-1 levels. IGF-1 causes autophosphorylation when its receptor binds to it, which activates PI3K. PI3K then phosphorylates and activates AKT, which in turn phosphorylates and activates mTOR while suppressing FOXO. This complete signaling cascade is influenced by CR, which decreases glucose levels. Studies have demonstrated that prolonging the life span of species such as yeast, worms, flies, and mice can be achieved by decreasing mTOR signaling (Johnson et al., 2013). Longevity was increased in mice given the mTOR inhibitor rapamycin. Since rapamycin reduces glucose tolerance and doesn't mimic the effects of dietary restriction on insulin, IGF-1, or leptin levels, the exact mechanism is yet unknown (Miller et al., 2014). FOXO transcription factors play a critical role in pathways related to longevity that include antioxidant activity, stress response, and cellular processes like autophagy, apoptosis, and proliferation (Martins et al., 2016), which also play a role in extending lifespan. Reduced insulin/IGF-1-like signaling in *C. elegans* increases lifespan and stress tolerance via blocking the FOXO protein DAF-16 and resulting in the nuclear build-up of SKN-1, a process that is independent of DAF-16 inhibition and controlled by AKT1/2 and SGK-1 phosphorylation (Tullet et al., 2008). CR has been shown to change the transcriptional and post-transcriptional levels of genes associated with the PI3K/AKT/FOXO pathway in both humans and rats. Notably, certain polymorphisms in the FOXO3 gene have been linked to increased lifespan and a reduced risk of mortality from both overall and coronary heart disease (Willcox et al., 2016). Furthermore, CR was able to improve several pathways in both humans and rats, including mitochondrial function, oxidative phosphorylation, muscular contraction, glycolysis, and gluconeogenesis. Conversely, pathways linked to the “aging brain” and insulin/IGF-1 signaling became less active (Mercken et al., 2013a).

#### 5.2.2 Role of miRNA as modulators of nutrient sensing pathway

Recent studies indicate that miRNAs have a notable impact on various aspects of aging, such as the disturbance of nutrient-sensing pathways, weakening of the immune system, cellular damage, and the onset of age-related diseases (López-Otín et al., 2013). The miRNA let-7, which targets the IGF1 receptor and mTOR among other components of the IGF1 pathway, has an impact on the IGF1/PI3K/AKT/mTOR pathway (Jung and Suh, 2014). Myoblast research has demonstrated that miR-432 inhibits myoblast development and proliferation by interfering with the PI3K/AKT/mTOR pathway (Chartoumpekis et al., 2012; Ma et al., 2017). Diet has been found to modulate this miRNA; for instance, miR-432 is downregulated in the adipose tissue of mice on a high-fat diet (HFD) (Chartoumpekis et al., 2012). Age-related decreases in miR-17-92 cause more DNA damage and oxidative stress (Grillari et al., 2010). MiRNAs also affect the AMPK/Sirtuins/PGC1-1 pathway, with let-7 controlling the production of SIRT1 in human biliary epithelial cells (Xie et al., 2014). miR-217 downregulates SIRT1, impacting endothelial cell aging through the silent information regulator 1 (SIR1) (Figure 4) (Menghini et al., 2009). Additionally, miR-133 inhibits AMPK expression, targeting this pathway at multiple points. Kurylowicz et al. observed in humans that a decrease in SIRT1 is associated with higher levels of miR-22-3p in obese individuals, while an increase in SIRT7 is linked to lower miR-125a-5p levels in thin individuals (Kurylowicz et al., 2016). Studies using *in vitro* and *in vivo* models have demonstrated that miR-16 reduces cell proliferation by targeting the IGF1R (Chen et al., 2013). Additionally, miR-144 plays a crucial role in regulating key pathways. It controls the IGF-1/PI3K/AKT pathway by targeting PTEN (Zhang et al., 2013) and IRS1 (Wu et al., 2016), and also directly influences mTOR (Xiang et al., 2016) and AMPK (Xiang et al., 2016). Olivieri et al. observed reduced levels of miR-182, miR-223, and miR-142-3p in the skeletal muscle of postmenopausal women. These miRNAs are involved in the regulation of IGF-1R and FOXO3A expression and activate the insulin/IGF-1 pathway by promoting the phosphorylation of AKT and mTOR (Olivieri et al., 2014). Notably, miR-142-3p was found to increase in the adipose tissue of mice on a HFD (Chartoumpekis et al., 2012), and a similar upregulation of miR-182 was observed in the liver of mice on an HFD (Tessitore et al., 2016).

Although the exact mechanisms through which CR prolongs lifespan remain unclear, miRNAs are assumed to be crucial. In rat studies, CR led to a decrease in miR-144 expression in cerebromicrovascular endothelial cells, which helps maintain NRF2 levels, a key regulator of cellular defense against oxidants (Csiszar et al., 2014). In rhesus monkeys aged rhesus, miR-144, which is typically upregulated in skeletal muscle with age, showed reduced upregulation. The same study found that other age-related miRNAs, which regulate the PI3K/AKT pathway and SIRT1, were significantly increased in older monkeys undergoing CR. Additionally, the role of miR-221, which is downregulated by both CR (Mercken et al., 2013b) and polyunsaturated fatty acids (Ortega et al., 2015), is noteworthy.

In contrast, the regulation miR-145 doesn't appear to be affected by CR, though it is increased by a HFD (Sangiao-Alvarellos et al., 2014). It is noteworthy to acknowledge the ongoing debate regarding alternative dietary approaches to promote longevity, such as protein restriction, which has the potential to alter the expression of specific miRNAs. Furthermore, it has been observed that a low-protein diet reduces the expression of miR-124a in the pancreatic islets of pregnant rats (de Siqueira et al., 2018), whereas, in overweight or obese males, a high-protein diet decreased HDL-associated miR-223 levels (Tabet et al., 2016). These findings suggest that different miRNAs can be affected by dietary interventions associated with longevity, such as CR or protein restriction. However, the precise consequences of these changes on extending lifespan still require further investigation to be fully elucidated.

#### 5.2.3 Nutraceuticals: regulation of miRNAs

Research has established a connection between typical neural development and the onset of neural disorders, which arises from a complex interplay between genetic factors and environmental influences, including nutrition. The role of diet in maintaining brain health throughout life is well-documented, with research indicating that what we eat affects neuron development, regeneration, function, and the maintenance of neural networks (Maher, 2000; Dauncey, 2009; Nurk et al., 2009; Gomez-Pinilla and Nguyen, 2012; Milte et al., 2012; Sinn et al., 2012). Interestingly, nutrition is one of the factors that can control gene expression without altering the DNA sequence itself. Recent focus has shifted to the role of miRNAs in both development and disease, including how their expression is influenced by nutrition. Although there has been extensive exploration of the role of nutrition in nervous system development and related issues like aging and neurodegenerative disorders, studies on the regulation of miRNAs in brain pathology by nutraceuticals are relatively sparse (Li D. et al., 2018). This review will cover a few existing studies on how nutraceuticals can regulate miRNAs associated with brain pathologies, including cancers and neurodegenerative disorders.

##### 5.2.3.1 Resveratrol

The primary mechanism by which resveratrol reduces proinflammatory lipid mediators is via inhibiting cyclooxygenase 1 and 2 (COX-1 and COX-2), as well as by reducing the mobility and activity of NF-κB’s p65 subunit when stimulated by TNF. This leads to reducing the levels of pro-inflammatory cytokines like TNF-alpha, IL-1 beta, and IL-6 (Latruffe et al., 2015). Additionally, resveratrol inhibits JNK and its upstream kinase MEK activation, which is advantageous for AP-1 activity suppression. Tili et al., 2010 demonstrated in human monocytes (THP-1), resveratrol could modulate AP-1 through epigenetic means by upregulating anti-inflammatory miR-663, targeting JunB and JunD, and downregulating pro-inflammatory miR-155. Song et al., 2016 further showed that longer exposure to resveratrol increased miR-Let7A expression in THP-1 cells, leading to reduced TNF-α and IL-6.

In a human study, researchers conducted a randomized, placebo-controlled trial involving 35 men with type-2 diabetes and hypertension. Over a year, participants were given capsules containing either a placebo (maltodextrin), grape extract supplemented with over 8 mg of resveratrol (GE-RES), or grape extract without resveratrol (GE). Compared to the control group, the GE-RES group showed alterations in miRNA associated with inflammation: miR-21, −181b, −663, and −30c2 levels increased, while miR-155 and −34a levels decreased. This suggests that resveratrol’s modulation of miRNAs might provide a protective effect against physiological variations (Tomé-Carneiro et al., 2013).

##### 5.2.3.2 Vitamins

Several vitamins have been shown to affect immunity and contribute to the prevention of disease. A good example of this is vitamin D, which can regulate the transcription of miRNA genes through its active form, dihydroxy vitamin D (1,25(OH)2D). This active form binds to the transcription factor vitamin D receptor (VDR) and influences the stability of miRNA or the genes responsible for miRNA processing (such as Drosha and Dicer) to enhance the maturity of miRNAs (Dambal et al., 2017; Zeljic et al., 2017).

Vitamin D inhibits inflammation through its effects on miRNAs, particularly miR-155, which has an antagonistic relationship with the vitamin D receptor (VDR). In patients with primary biliary cholangitis, the SOCS1 protein in the liver and PBMCs is correlated with the levels of miR-155 (Kempinska-Podhorodecka et al., 2017). Furthermore, vitamin D decreases miR-146a, miR-150, and miR-155 in murine adipocytes to suppress NF-κB signaling, which in turn affects p65 and IκB phosphorylation (Karkeni et al., 2018). VDR activators, like calcitriol and paricalcitol, suppress miR-29b and miR-30c in nephrectomized rats, impacting genes like COL1A1, MMP-2, and CTGF, thus reducing cardiac fibrosis (Panizo et al., 2017). A 12-week treatment was shown to reduce inflammatory and atherosclerotic cytokines and miRNAs in patients with moderate chronic renal disease (Mansouri et al., 2017). Moreover, the research indicates that supplementing with vitamin D enhances the presence of miRNAs that suppress tumors in the prostate tissue of patients (Giangreco et al., 2013)

Vitamin A derivatives, such as various forms of retinoic acid, are known to control gene expression in both normal and disease conditions (Perri et al., 2017). In an experiment with mouse embryonic stem cells, exposure to retinoic acid resulted in the upregulation of 31 miRNAs and the downregulation of 175 miRNAs. The decreased miRNAs miR-200b and miR-200c were distinguished for their noteworthy ability to significantly upregulate the expression of two pluripotent genes that are critical for development and epithelial phenotyping: Oct4 and Nanog (Zhang J. et al., 2015). The expression of miR-10a and the Retinoic Acid Receptor Beta (RARβ) is significantly increased by retinoic acid (RA) in two breast cancer cell lines, T47D and SK-BR-3. Due to their associations with tumor suppression in breast tissue samples, reduced expression of RARβ and miR-10a has been related to breast cancer (Khan et al., 2015).

##### 5.2.3.3 Curcumin

Besides inflammation recent research has revealed that curcumin can influence certain miRNAs in various cancer cell types through its impact on the inflammatory response. Kronski et al. (Kronski et al., 2014) found that treating breast cancer cells with curcumin leads to an increase in miR-181b levels, which in turn reduces the production of pro-inflammatory cytokines CXCL1 and −2. This reduction contributes to a decreased metastatic potential in these cells. Additionally, research on breast stromal fibroblasts has demonstrated that the tumor-suppressing protein p16INK4A, which is regulated by miR-146b-5p, lowers the carcinogenic effects of these cells by regulating IL-6 production and secretion. This particular miR targets a specific sequence in the IL-6 3′UTR. Treatment with curcumin may increase the levels of p16INK4A and miR-146b-5p, which could then result in the reduction of IL-6 (Al-Ansari and Aboussekhra, 2015).

##### 5.2.3.4 Quercetin

Quercetin’s role in reducing inflammation is connected to its capability to suppress both the ERK and JNK proteins, as well as their phosphorylated forms, and to lower the production of TNF-α induced by these proteins.

In research by Boesch-Saadatmandi et al. (Boesch-Saadatmandi et al., 2012), it was revealed that female mice consuming a diet high in quercetin (2 mg/g) showed notably increased levels of hepatic miR-125b and miR-122 compared to those on a regular diet. miR-125b is known for its capacity to diminish inflammation, whereas miR-122 plays a key role in the control of lipid metabolism. In a separate study focusing on the influence of quercetin and its main metabolites on miR-155, researchers observed that the use of quercetin and isorhamnetin on LPS-stimulated macrophages resulted in a reduction in miR-155 expression. This effect could suggest a potential mechanism by which this polyphenol hinders NF-κB activation, consequently contributing to the attenuation of inflammation (Boesch-Saadatmandi et al., 2011).

Diets high in saturated fatty acids, particularly from high-fat foods, are linked to health issues like gut imbalance, low-grade inflammation, and insulin resistance (Cani et al., 2007; O'Keefe et al., 2008; Ruiz-Núñez et al., 2013; Martinez et al., 2017). Certain saturated fats, like lauric and palmitic acids, trigger inflammation in various cells by activating specific receptors, leading to increased inflammatory gene expression (Wong et al., 2009; Wang Y. et al., 2017). Recent studies also suggest that saturated fats can influence these processes through epigenetic changes, including the action of miRNAs.

##### 5.2.3.5 Polyunsaturated fatty acids

Lipid mediators formed from essential fatty acids, such as thromboxanes, lipoxins, resolvins, and leukotrienes, are a few examples of how polyunsaturated fatty acids (PUFA) might affect the inflammatory response. Studies have demonstrated that these lipid mediators can control the expression of genes by means of miRNAs (Fredman et al., 2012; Krishnamoorthy et al., 2012; Visioli et al., 2012). In a study by Zheng et al., 2015 rats that were about 3 weeks old were given a Western-style diet for a week. After that, the rats were divided into three groups, one of which received an omega-3 PUFA diet (a combination of EPA and DHA), another of which received an omega-6 PUFA diet (linoleic acid), and a control group. After 16 weeks, in comparison to the control group, the omega-3 group showed a reduction in subcutaneous fat and pro-inflammatory cytokines. Unlike the omega-6 and control groups, this group’s levels of miRNAs (rno-miR-19b-3p, −146b-5p, and −183-5p) were decreased. These miRNAs were found to suppress genes involved in inflammatory pathways.

Additionally, during 8 weeks, thirty healthy participants in a human trial ate 30 g of polyunsaturated fatty acid-rich almonds and pecans every day. Eleven miRNAs were altered in their plasma as a result: levels of miR-192, miR-486-5p, miR-19b, miR-106a, miR-130b, miR-18a, and miR-769-5p were elevated and levels of miR-328, miR-330-3p, miR-221, and miR-125a-5p were lowered. Notably, favorable changes in the levels of the plasma protein C-reactive were associated with miR-221 and miR-130b (Ortega et al., 2015).

## 6 Implications for prevention and treatment of age-related diseases

### 6.1 miRNA involvement in the therapeutic impact of nutraceuticals on aging and age-related diseases

Aging and age-related diseases occur due to multi-faceted pathologic incidences. One of these pathologic incidences is the chronological increase in senescent cells (SCs) which undergo irreversible cell cycle arrest, and simultaneously increase the release of various chemicals that stimulate inflammation and oxidative stress signaling pathways. This SCs phenotype is identified as a senescence-associated secretory phenotype (SASP) (Prasnikar et al., 2021). Therefore, foods high in polyphenols and other bioactive compounds, which have anti-inflammatory and antioxidant properties, may be used as “anti-senescence” or “senolytic” foods in a nutraceutical strategy for enhanced cellular longevity and better aging (Gurau et al., 2018). Indeed, nutraceuticals rich in polyphenols have shown excellent results in many age-related diseases owing to their antioxidant and anti-inflammatory effects (Zhor et al., 2023).

Epigenetic dysregulation is a part of the aging process, and many nutraceuticals are regarded as promising candidates to counteract these age-related epigenetic changes (Bacalini et al., 2014). Likewise, changes in miRNA expression play a prominent role in regulating the mRNA expression of genes involved in the shaping of aging and age-related diseases (Matai and Slack, 2023a). Therefore, it’s expected that diets or nutraceuticals that control specific miRNA expression can enhance healthy aging or halt the progression of age-related diseases. So far, it has been reported that dietary intake of micronutrients, macronutrients, vitamins, minerals, flavonoids, and polyphenols can modulate the expression of various miRNAs (Dimmeler and Nicotera, 2013; Milenkovic et al., 2013; Beckett et al., 2014; Yu et al., 2021). However, future research will need to determine whether dietary manipulation of miRNAs contributes to the prevention or treatment of age-related illnesses.

As we age, we experience a low-grade systemic inflammation, a condition that has been previously termed “inflammaging.” This condition is marked by an increase in the levels of pro-inflammatory cytokines (mostly TNF-α and IL-6) and circulating acute-phase proteins (Franceschi et al., 2000; Franceschi, 2007). Frailty and the onset and advancement of age-related illnesses, such as cancer, osteoporosis, neurodegenerative diseases, atherosclerotic cardiovascular diseases, and type 2 diabetes mellitus are linked to this inflammaging phenomenon (Vasto et al., 2007; Cevenini et al., 2013). Numerous research provided evidence in favor of the theory that achieving extreme longevity likely necessitates a unique control of inflamma-miRs gene expression (ElSharawy et al., 2012; Olivieri et al., 2013). Quercetin, a plant flavonol, shielded CCD-18Co myofibroblasts from reactive oxygen species (ROS) and prevented inflammation by enhancing the activity of antioxidant enzymes and stimulating the upregulation of miR-146a, a negative regulator of pro-inflammatory nuclear factor kappa B (NFκB) activation (Noratto et al., 2011b).

**FIGURE 5:**
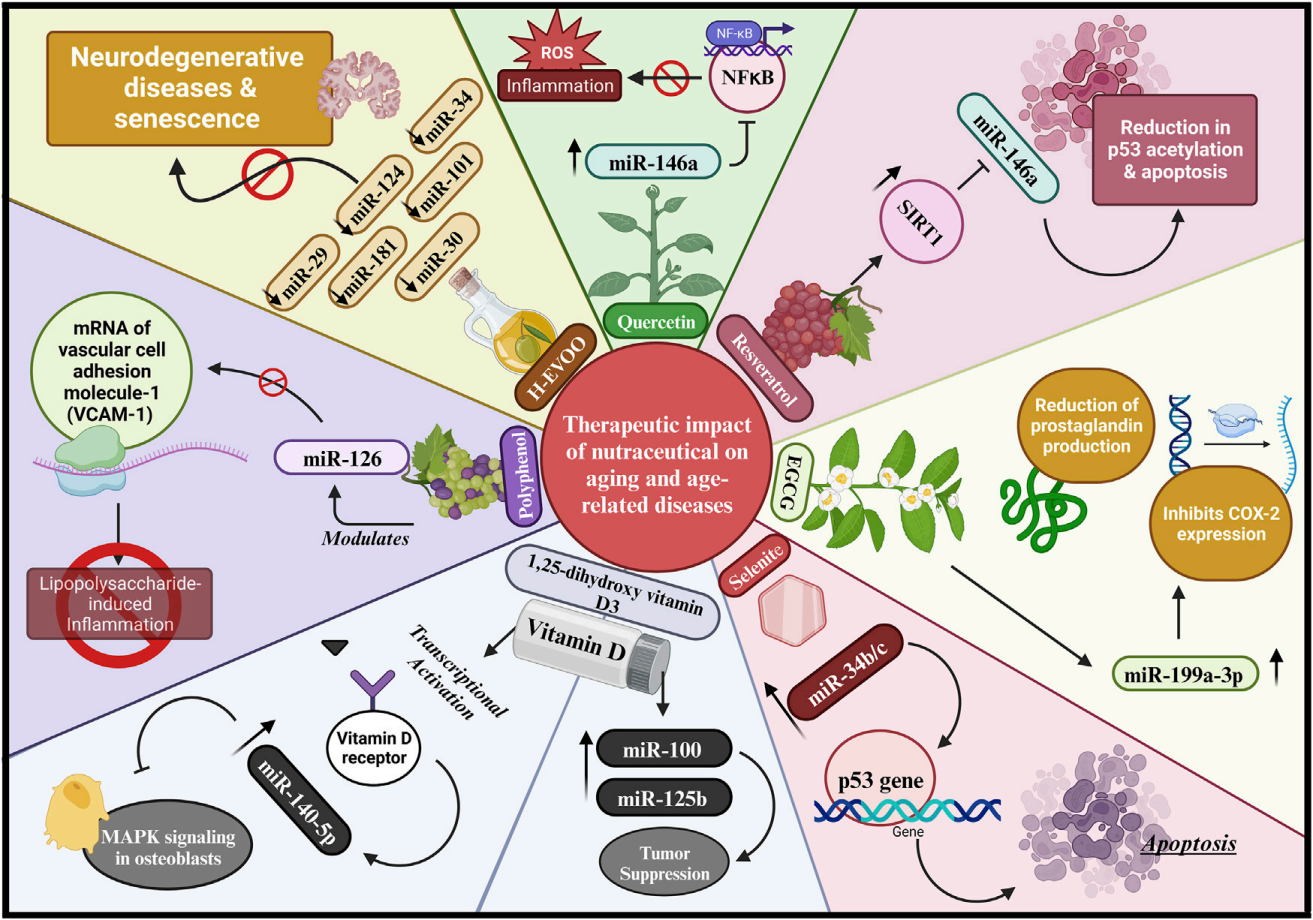
miRNA involvement in the therapeutic impact of nutraceuticals on aging and age-related diseases. Nutraceuticals rich in polyphenols and bioactive compounds exhibit anti-inflammatory and antioxidant effects, potentially enhancing cellular longevity and mitigating age-related changes. These compounds can influence miRNA expression, offering promising strategies for promoting healthy aging and preventing or treating age-related diseases. COX-2, cyclooxygenase 2; EGCG, epigallocatechin-3-gallate; H-EVOO, high-extra virgin olive oil; MAPKs, mitogen-activated protein kinases; NF-κB, nuclear factor kappa beta; ROS, reactive oxygen species; SIRT1, sirtuin 1.

Changes in miRNA expression were evident in brain aging and neurodegenerative diseases. Whether these changes in miRNA expression are the cause or consequence of brain aging is still a riddle (Somel et al., 2010). In the study of Luceri et al. (Luceri et al., 2017b), mice fed extra-virgin olive oil (EVOO) rich in phenols (H-EVOO) for 6 months demonstrated improvements in their motor and cognitive functions when compared to controls given the same olive oil but devoid of phenolics (L-EVOO). Interestingly, mice fed L-EVOO demonstrated a substantial modification in the expression of 175 miRNA, unlike mice fed H-EVOO which showed alterations in the expression of only 20 miRNAs compared to young mice. These results demonstrate the capacity of phenolic-rich olive oil to mitigate age-related modifications in miRNA expression. Moreover, miR-29, miR-30, miR-34, miR-101, miR-124, and miR-181, which have been linked to neurodegenerative diseases or senescence (Dimmeler and Nicotera, 2013), were among the miRNAs that were either low or not expressed in the cortex of young and H-EVOO-fed mice and strongly expressed in mice fed L-EVOO (Figure 5).

As stated by Sir William Osler in his historical textbook in 1898: *“Longevity is a vascular question, which has been well expressed in the axiom that man is only as old as his arteries”* (COOK, 1908). Indeed, aging is intricately linked to the development of CVDs. Growing older is the primary risk factor for vascular endothelial dysfunction, which raises the possibility of CVDs, one of the leading causes of death in developed nations (Gaidai et al., 2023). These CVDs are mostly attributable to the sedentary lifestyle in the developed world, characterized by a diet rich in saturated fats and sugars (Kirkpatrick and Maki, 2021). On the contrary, a reduction in the onset of CVDs could be attributable to health-promoting dietary patterns, such as the Mediterranean diet rich in nutraceuticals (Accardi et al., 2016). An *in vitro* study conducted by Noratto et al. (2011a) showed that treatment of lipopolysaccharide-induced inflammation in human umbilical vein endothelial Cells (HUVEC) with a polyphenol extracted from grapes reduced the mRNA expression of vascular cell adhesion molecule-1 (VCAM-1) as a result of modulating the expression of miR-126.

Bone health deteriorates and the incidence of osteoporosis increases with increased age (Pignolo et al., 2021). Vitamin D is a key regulator of bone health by promoting calcium absorption, and its active form, 1,25-dihydroxyvitamin D (1,25-(OH)_2_-D), acts as a steroid hormone and exerts pleiotropic effects (Veldurthy et al., 2016). In a previous study by Jiao et al., 2020, the beneficial role of miR-140-5p in bone health was disclosed. Results revealed that miR-140-5p promoted osteogenic differentiation and led to a significant increase in osteocalcin expression, bone mineral density, and bone mass in addition to enhancing bone healing of miR-140-5p-transgenic mice with fracture. In the study conducted by Luo et al., 2018, a positive correlation between miR-140-5p and 1,25-(OH)_2_-D was revealed. In this study, miR-140-5p was upregulated in 2 types of murine osteoblasts treated with 1,25-(OH)_2_-D. The enhanced expression of miR-140-5p by 1,25-(OH)_2_D3 occurred through transcriptional activation of the vitamin D receptor and this activated vitamin D/vitamin D receptor/miR-140-5p axis led to a downstream inhibition of MAPK signaling in osteoblasts (Figure 5).

Cancer is regarded as one of the age-related diseases due to the build-up of mutations throughout life (Laconi et al., 2020). miR-100 and miR-125b were shown to have tumor suppressor effects and their expression levels were found to be reduced in several cancers. The study conducted by Giangreco et al., 2013 revealed another health benefit for 1,25-dihydroxy vitamin D3 in prostate cancer patients, in which vitamin D3 supplementation managed to enhance the expression of these tumor-suppressive miRNAs. This suggests that miRNAs may be important physiological mediators of vitamin D3 action in the early detection and prevention of prostate cancer.

All-trans-retinoic acid treatment upregulates miR-29a and miR-142-3p. In patients with acute myeloid leukemia (AML) and healthy controls, enhanced expression of either miRNA-induced myeloid differentiation. This shows that miR-29a and miR-142-3p are important regulators of healthy myeloid differentiation and that the development of AML is influenced by their decreased expression (Wang et al., 2012). Selenite is a nutraceutical and dietary form of selenium. It was shown that treatment of human prostate cancer cells (LNCaP) with selenite upregulated the expression of p53 and miR-34b/c, where miR-34 is known to target the p53 gene, and induction of p53 enhances apoptosis. This indicates that selenite may be beneficial for both the prevention and treatment of human prostate cancer (Sarveswaran et al., 2010).

Resveratrol, found in red grapes, is a popular bioactive compound that was extensively studied during the last 2 decades and found to have a myriad of protective effects against a wide variety of diseases (Meng et al., 2020). Adding to its well-known benefits, resveratrol can be protective against age-related problems such as hearing loss in which miR-34a was found to be involved in resveratrol’s protective mechanism. In the study of Xiong et al., 2015, miR-34a expression, p53 acetylation, and apoptosis were found to be enhanced in the cochlea of C57BL/6 mice with aging, however, an age-linked reduction in SIRT1 was noted. In the inner ear HEI-OC1 cell line, an inverse relation was revealed between miR-34a and SIRT1 expression; where the overexpression of miR-34a inhibited SIRT1 expression, which led to a rise in p53 acetylation and apoptosis. Inversely, silencing of miR-34a led to a significant increase in SIRT1 expression, and consequent reduction in p53 acetylation, and apoptosis. Resveratrol, being a SIRT1 activator, following 2-month administration, significantly attenuated miR-34a expression, which led to a significant reduction in hearing loss and hair cell loss in C57BL/6 mice (Figure 5).

Epigallocatechin-3-O-gallate (EGCG) is the most abundant and active polyphenol in green tea. The study of Rasheed et al., 2016 investigated the effect of EGCG in an *in vitro* model of osteoarthritis, one of the age-related chronic diseases. The study revealed that EGCG inhibited COX2 expression and prostaglandin E2 (PGE2) production via upregulating miR-199a-3p expression in an IL-1β-stimulated human osteoarthritis chondrocytes.

Notably, the majority of the data supporting the modification of miRNA by nutraceuticals in aging comes from cell lines and some of this data comes from *in vivo* pre-clinical studies. Translational research that can advance these studies from bench-to bedside or from pre-clinical to clinical studies in older adults will help determine if nutraceutical manipulation of miRNAs can improve healthy aging or halt the occurrence or progression of age-related diseases.

### 6.2 Strategies for utilizing miRNA-based interventions in age-related diseases

miRNAs have a crucial function in regulating gene expression post-transcription. They have emerged as important regulators of cellular functions and can be altered by dietary factors. Specific miRNAs selectively target genes responsible for encoding proteins and enzymes involved in pathways that sense nutrition. This targeting has the potential to greatly impact the regulation of the aging process (Lai et al., 2019). Several studies have demonstrated that diet and nutraceuticals can potentially control the expression of miRNAs linked to aging, neurodegenerative disorders, and brain tumors. Nutrients possess the capacity to control the amounts of miRNA, which plays a pivotal role in influencing gene expression. Therefore, the control of miRNA aging through nutrition and nutraceuticals holds great potential for treating age-related diseases and improving overall healthy aging.

The approaches for employing miRNA-based interventions in age-related diseases encompass the identification of disease-linked miRNAs, the manipulation of miRNA expression through nutrition and nutraceuticals, the creation of miRNA-based therapeutics, and the investigation of miRNAs as biomarkers for age-related diseases. These methods show potential for creating new strategies to encourage healthy aging and tackle age-related ailments (Lai et al., 2019).

Studies have demonstrated that different miRNAs play a role in the regulation of age-related illnesses, including AD, age-related macular degeneration, neurological disorders, and cancer (Natoli and Fernando, 2018; Eshkoor et al., 2022). Studies have shown that nutritional components and nutraceuticals can control the activity of miRNAs that have a role in age-related disorders. This suggests that they could be used as potential treatments (Ghosh et al., 2021).

miRNAs have been recognized as promising candidates for therapeutic intervention in age-related macular degeneration and other age-related ailments. Several miRNA-based medicines are currently undergoing Phase 2 clinical studies, indicating the efficacy of miRNAs as therapeutic targets (Natoli and Fernando, 2018).

This topic investigates the impact of nutrition on the alteration of gene expression through epigenetic mechanisms associated with miRNAs. This study assesses the impact of nutrients and bioactive chemicals on the expression of miRNA, which may have consequences for regulating inflammation and chronic diseases (Quintanilha et al., 2017a). Specific miRNAs have been proposed as prospective biomarkers, diagnostic tools, or desirable targets for the management and treatment of age-related illnesses, such as age-related macular degeneration (Cruz-Aguilar et al., 2023).

## 7 Nutraceuticals’ impact on aging and related diseases

In a previously reported systematic review, 200 adults were assessed on their use of different nutraceuticals. It was found that using nutraceuticals has improved mitochondrial oxidative capacity and bioenergetics (Lippi et al., 2022). A balanced diet supplemented with limonene controlled inflammation in elderly individuals decreasing fibrinogen and inflammatory markers (Ostan et al., 2016). In a retrospective study, administration of folic acid and vitamin B was linked to speed of processing, recall, identification, and verbal capability in middle-aged and elderly women (Bryan et al., 2002). Moreover, nutraceuticals containing fish oil and coenzyme Q10 are associated with reduced risk and retard the progression of Parkinson’s disease (Mischley et al., 2017). Further, a polyphenolics-rich diet inhibits lipid peroxidation in the CNS reducing aging-related diseases in a double-blinded clinical study (Arcusa et al., 2023).

## 8 Conclusion

MiRNAs hold great potential as a field of study for comprehending the molecular pathways that drive aging and age-related illnesses. The data given in this review emphasizes the possible influence of dietary variables and nutraceuticals in regulating miRNA expression and function. Existing research indicates that certain macronutrients, micronutrients, trace minerals, and nutraceuticals might impact miRNA profiles. Nevertheless, the majority of the existing research has been carried out in laboratory settings, and further investigations are required to clarify the impact of dietary miRNA manipulation on living organisms, including animal models and human patients. Subsequent studies should prioritize the examination of the specific mechanisms via which dietary variables and nutraceuticals regulate miRNA, as well as the exploration of the therapeutic possibilities of miRNA-based therapies in age-related disorders. By enhancing our comprehension of the intricate nutritional coordination facilitated by miRNAs, we might potentially establish novel approaches to encourage the process of healthy aging and counteract age-related disorders.

## Figures and Tables

**Table:** 

**AD**	Alzheimer’s disease
**ALK**	Anaplastic lymphoma kinase
**AMD**	age-related macular degeneration
**AML**	acute myeloid leukemia
**AMPKs**	AMP-activated protein kinases
**COX-** **1**	cyclooxygenase 1
**COX-** **2**	cyclooxygenase 2
**CR**	Caloric restriction
**CVDs**	cardiovascular diseases
**FOX**	forkhead box
**HFD**	high-fat diet
**HUVEC**	Human Umbilical Vein Endothelial Cells
**IGF-1**	insulin/insulin-like growth factor**-**1
**IIS**	Insulin/IGF-1 signaling
**MAPKs**	mitogen-activated protein kinases
**miRNAs**	MicroRNAs
**mRNA**	messenger RNA
**NAMPT**	nicotinamide phosphoribosyltransferase
**ncRNAs**	non-coding RNAs
**NMN**	Nicotinamide mononucleotide
**PDK**	phosphatidylinositol (PtdIns)-dependent kinase
**PGE2**	prostaglandin E2
**pri-miRNA**	primary miRNA
**PTEN**	protein phosphatase and tensin homolog deleted in chromosome 10
**PUFA**	polyunsaturated fatty acids
**RTKs**	receptor tyrosine kinases
**RYK**	receptor-Like Tyrosine Kinase
**SASP**	senescence-associated secretory phenotype
**SCs**	senescent cells
**SIR1**	silent information regulator 1
**snoRNAs**	Small nucleolar RNAs
**TMAO**	Trimethylamine-N-oxide
**TSC**	tuberous sclerosis complex
**VCAM-1**	vascular cell adhesion molecule**-**1
**VDR**	vitaminD receptor
